# UVA influenced the SIRT1‐miR‐27a‐5p‐SMAD2‐MMP1/COL1/BCL2 axis in human skin primary fibroblasts

**DOI:** 10.1111/jcmm.15610

**Published:** 2020-08-13

**Authors:** Shi‐Bin Jiang, Yan‐Song Lu, Tao Liu, Liang‐Man Li, He‐Xiao Wang, Yan Wu, Xing‐Hua Gao, Hong‐duo Chen

**Affiliations:** ^1^ Department of Dermatology The First Hospital of China Medical University Shenyang China; ^2^ Department of Urinary Surgery The First Hospital of China Medical University Shenyang China; ^3^ Department of Orthopedics The First Hospital of China Medical University Shenyang China

**Keywords:** human skin primary fibroblasts, miR‐27a‐5p, SIRT1, UVA

## Abstract

Both SIRT1 and UVA radiation are involved in cellular damage processes such as apoptosis, senescence and ageing. MicroRNAs (miRNAs) have been reported to be closely related to UV radiation, as well as to SIRT1. In this study, we investigated the connections among SIRT1, UVA and miRNA in human skin primary fibroblasts. Our results showed that UVA altered the protein level of SIRT1 in a time point–dependent manner. Using miRNA microarray, bioinformatics analysis, we found that knocking down SIRT1 could cause up‐regulation of miR‐27a‐5p and the latter could down‐regulate SMAD2, and these results were verified by qRT‐PCR or Western blot. Furthermore, UVA radiation (5 J/cm^2^), knocking down SIRT1 or overexpression of miR‐27a‐5p led to increased expression of MMP1, and decreased expressions of COL1 and BCL2. We also found additive impacts on MMP1, COL1 and BCL2 under the combination of UVA radiation + Sirtinol (SIRT1 inhibitor), or UVA radiation + miR‐27a‐5p mimic. SIRT1 activator resveratrol could reverse damage changes caused by UVA radiation. Besides, absent of SIRT1 or overexpression of miR‐27a‐5p increased cell apoptosis and induced cell arrest in G2/M phase. Taken together, these results demonstrated that UVA could influence a novel SIRT1‐miR‐27a‐5p‐SMAD2‐MMP1/COL1/BCL2 axis in skin primary fibroblasts, and may provide potential therapeutic targets for UVA‐induced skin damage.

## 1 INTRODUCTION

Ultraviolet A (UVA) penetrates through the epidermis into the dermis and causes detrimentally photochemical reactions. It leads to the remodelling of extracellular matrix (ECM) by increasing matrix metalloproteinase 1 (MMP1), reducing structural collagen or changing cell signalling and phenotypes.^1^, ^2^ As a potent driver of oxidative DNA damage in skin cells, UVA causes gene mutations by oxidizing guanine at the 8th position to produce 8‐hydroxy‐2’‐deoxyguanine and ultimately generation of reactive oxygen species (ROS).^3^, ^4^ UVA‐induced oxidative stress in epidermal compartment prompts stem cell damage which in turn activates cell senescence, a form of irreversible growth arrest and apoptotic resistance that represents one of the main causes of photoageing.^5^, ^6^ Photoageing is characterized by decreased expression of collagens and increased expression of MMPs, and is potentially related to skin cancer.^7^, ^8^

Being a class of histone deacetylases, sirtuins are associated with mammalian metabolism and lifespan of lower organisms.^9^, ^10^ Silent information regulator 1 (SIRT1) is the homology of silent information regulator 2 (Sir2) that has been previously extensively studied. SIRT1 regulates expression and activity of proteins, such as PGC‐1α, p53 and FOXO1 via deacetylating histone and non‐histone proteins in a nicotinamide adenine dinucleotide + dependent way.^11^, ^12^ SIRT1 is involved in the regulation of numerous biological processes, such as metabolism, cell cycle, DNA repair, cell survival and ageing.^9^, ^10^ SIRT1 also participates in the process of resisting oxidative stress and eliminating ROS. Csiszar showed that caloric restriction (CR) diet could significantly decrease the production of TNF‐α–induced ROS, inhibit the activity of NF‐κB and reduce the oxidative stress responses, and these effects could be significantly weakened in SIRT1‐knockdown rat models.^13^ The overlapping between SIRT1 involved‐protection mechanisms and UVA induced‐damage processes indicated a close relationship between SIRT1 and UVA.

MicroRNAs (miRNAs) is a group of highly evolutionarily conserved non‐coding small RNA molecules (19‐24 nucleotides) that regulate gene expression at the post‐transcriptional level.^14^ MiRNAs have been reported to be closely related to UV radiation. For example, UV‐inducible miR‐16 down‐regulated CDC25a and regulated cell proliferation in human fibroblasts.^15^ Increase in miR‐22 may protect cells from UV‐induced apoptosis by repressing PTEN in HEK293T cells.^16^ Up‐regulation of miR‐125b induced by NF‐kappa B promoted cell survival upon UV radiation.^17^ There are also associations between miRNAs and SIRT1. Guan *et al* have reported that miR‐30a inhibited the proliferation, invasion and apoptosis of lung cancer cells by inhibiting SIRT1 in vivo and in vitro.^18^ Overexpression of miR‐221 has been found to reduce the protein abundance of SIRT1, and cause inflammation and insulin resistance in differentiated 3T3‐L1 cells.^19^

As these evidences have suggested certain potential connections among SIRT1, UVA and miRNA, we hypothesized that SIRT1 regulates miRNA medicating downstream pathways/biomarkers, and thus, they play important roles in UVA‐induced skin damage. Therefore, we silenced SIRT1 to identify potential target miRNA and its’ downstream markers in human skin primary fibroblasts, under UVA irradiation circumstance or not. We were sought to find a novel SIRT1‐miRNA regulating axis to provide potential therapeutic targets for UVA‐induced skin damage.

## 2 MATERIALS AND METHODS

### 2.1 Cell culture

The experiment complied with the ethical standards of the Institutional Medical Ethics and Human Research Committee. Ethics number: AF‐SOP‐07‐1.0‐01. Skin primary fibroblasts were extracted from discarded foreskin tissues of three healthy individuals at ages of 17, 24 and 25 years old that were obtained from department of urology surgery of the first hospital of China Medical University. Cells were maintained with Dulbecco’ s modified Eagle medium (HyClone) containing 10% foetal bovine serum (HyClone) and 1% penicillin‐streptomycin solution (BI, ISR), and cultured at 37°C in 5% CO_2_ humidified atmosphere.

### 2.2 UVA radiation

The cells 1 × 10^5^ were seeded per well in 6‐well plates. Fibroblasts were grown to 60%‐70% confluence in a 6‐well plate before UVA exposure. Fibroblasts were washed with PBS twice and covered with 500 μL of PBS per well in 6‐well plates before exposed to UVA radiation by UVA Therapy System (5 J/cm^2^ UVA, UV 801 KL, Waldmann, GER). The distance between the cells and the lamp was 15 cm. Fresh medium was then used to replace PBS and maintain the cell culture for subsequent experiments.

### 2.3 Immunofluorescence staining

Skin primary fibroblasts were fixed with 4% paraformaldehyde for 20 minutes, permeated with PBS containing 0.2% Triton X‐100 and 1% BSA for 5 minutes, and blocked with 1% BSA in PBS for 1 hour Cells were then incubated with rabbit anti‐vimentin (VIM) antibody (diluted 1:100, BOSTER) or mouse anti‐alpha smooth muscle actin (a‐SMA) antibody (diluted 1:100, Abcam) overnight at 4°C, and incubated with fluorescein‐conjugated goat anti‐rabbit IgG (H + L) (diluted 1:50, ZSGB‐BIO) or fluorescein‐conjugated goat anti‐mouse IgG (H + L) (diluted 1:50, ZSGB‐BIO) for 2 hours. Finally, cells were dyed with 4’‐6‐diamidino‐2‐phenylindole (DAPI) for nuclei for 10 minutes in dark and then observed under a fluorescence microscopy (Leica).

### 2.4 Transfections of SIRT1 small interfering RNAs, miRNA‐27a/432 mimic

SIRT1 small interfering RNAs (siRNAs), negative control no. 1 siRNA, miRNA‐27a/432 mimic and miRNA mimic negative control #1 were all purchased from Thermo Fisher Scientific. Twenty‐four hours after plating, when cells were at a confluency of 70%, 10 nM of siRNA or negative control no. 1 siRNA and 30 nM of miRNA‐27a/432 mimic or miRNA mimic negative control #1 were transfected into the cells using 5 μL of Lipofectamine RNAiMAX (Invitrogen) according to the instructions. After 72 hours of transfection, RNA and protein, and the transfection efficiency were confirmed by qRT‐PCR and Western blot, respectively. All experiments were tested at least three times.

### 2.5 Additions of resveratrol, SRT 1720 or Sirtinol

The resveratrol (RSV) and SRT 1720 were SIRT1 activators, and Sirtinol was SIRT1 inhibitor. When primary fibroblasts were at a confluency of 70%, cells were washed with PBS for three times and then were incubated with 10 μM of RSV (MCE), 2 μM SRT 1720 HCL (Selleck) or 10 μM Sirtinol (MCE) for 24 hours. The cells were harvested for subsequent experiments.

### 2.6 qRT‐PCR assay

Total RNAs were extracted from skin primary fibroblasts with miRNeasy mini kit (Qiagen). For mRNA, cDNA synthesis was performed using GoScript™ Reverse Transcription System (Promega), and then, the cDNA was subjected to real‐time PCR with GoScript™ qPCR Master Mix (Promega). Primers (BGI) used for PCR were as follows: SIRT1, 5’‐CGGAAACAATACCTCCACCTGA‐3’ (f), 5’‐GAAGTCTACAGCAAGGCGAGCA‐3’ (r); GAPDH, 5’‐GACAACTTTGGCATCGT‐3’ (f), 5’‐ATGCAGGGATGATGTTCTGG‐3’ (r). For miRNA, the expression levels were detected using TaqMan™ MicroRNA Reverse Transcription Kit (Thermo Scientific) and corresponding primers (Thermo Scientific) were as follows: mature hsa‐miR‐27a sequence, AGGGCUUAGCUGCUUGUGAGCA; mature hsa‐miR‐432 sequence, CUGGAUGGCUCCUCCAUGUCU; U6 snRNA sequence, GTGCTCGCTTCGGCAGCACATATACTAAAATTGGAACGATACAGAGAAGATTAGCATGGCCCCTGCGCAAGGATGACACGCAAATTCGTGAAGCGTTCCATATTTT. The PCR amplification was carried out by 7900HT fast real‐time PCR system (Applied Biosystems). Melt curve analysis was carried out to check the specificity of each primer‐pair. The 2^‐△△Ct^ was used to calculate relative gene expression. All experiments were tested at least three times.

### 2.7 Western blot analysis

Skin primary fibroblasts were harvested when cells were at a confluency of 80%‐90%. The protein content was measured with BCA protein assay kit (Beyotime). Denatured proteins (20 μg) were separated by 10% SDS‐PAGE and transferred to 0.45 μm PVDF membrane, blocked with TBST containing 5% skim milk or 5% BSA and incubated with rabbit anti‐SIRT1 antibody (1:1000, Abcam), rabbit anti‐Smad2/3 antibody (1:1000, CST), rabbit anti‐Smad4 antibody (1:1000, CST), rabbit anti‐YAP/TAZ antibody (1:1000, CST), rabbit anti‐MMP1 antibody (1:1000, ProteinTech Group), rabbit anti‐COL1 antibody (1:1000, Abcam), rabbit anti‐BCL2 antibody (1:1000, Abcam) and mouse anti‐GAPDH antibody (1:10 000, ProteinTech Group) at 4°C overnight. The membranes were incubated with HRP‐labelled goat anti‐rabbit IgG (H + L) (1:1000, Beyotime) or HRP‐labelled goat anti‐mouse IgG (H + L) (1:1000, Beyotime) for 1.5 hours at room temperature. The blots were visualized with enhanced ECL (Beyotime) following exposure to DNR MF‐ChemiBIS 2.0 (ISR). All experiments were tested at least three times.

### 2.8 Flow cytometry assay

For apoptosis analysis, cells were harvested and stained with 5 μL FITC Annexin V (BD Bioscience) and 10 μL PI (BD Bioscience) for 15 minutes in dark. For cell cycle analysis, cells were harvested, washed with cold PBS for three times and fixed with 1.5 mL of 75% ethanol at 4°C overnight. Cells were then stained with 3 μL RNaseA (Sigma) for 30 minutes at 37°C and 10 μL PI for 30 minutes on ice in dark. Flow cytometry analysis was performed by BD LSRFortessa instrument (BD Bioscience). ModiFit LT (version 3.3) was used to analyse the cell cycle regulation.

### 2.9 MiRNA microarrays

Total RNAs were extracted from three SIRT1‐silenced (si‐SIRT1) skin primary fibroblasts and three control fibroblasts. The potential miRNAs were detected by using TaqMan Array Human MicroRNA A + B Cards (Thermo Scientific). This microarray could quantify 754 human miRNAs (Table S1). Three endogenous controls (U6, RNA44 and RNA48) and one non‐human negative control were also included in this microarray. The experiment was performed following the manufacturer's instructions. In brief, each fill reservoir of the card was loaded with 100 μL of prepared PCR reaction mix, including 450μl PCR Master Mix, 6μl cDNA and 444μl Nuclease‐fee Water. Then, set up and run the real‐time PCR instrument (7900HT Fast Real‐Time PCR System). Experimental conditions were as follows: 94.5°C, 10 minutes, 1 cycle for enzyme activation; 97.0°C, 30 seconds, 40 cycles for denature; 59.7°C, 60 seconds, 40 cycles for anneal/extend.

### 2.10 Prediction of miRNA target genes

Eight bioinformatics tools, including miRanda, PicTar, PITA 6.0, RNA22 2.0, TargetScan 7.2, miRDB, miRWalk 2.0 and miRNAMap 1.0, were used to predict the potential target genes of differentially expressed miRNAs. Target genes were selected based on the criteria of being identified by more than four bioinformatics tools with statistical significance. The combined positions between miRNAs and potential target genes were predicted by TargetScan 7.2.

### 2.11 Bioinformatics analysis

Predicted target genes were analysed using Gene ontology (GO) enrichment analysis to identify three independent categories, that is biological process (BP), cellular component (CC) and molecular function (MF). Kyoto Encyclopedia of Genes and Genomes (KEGG) Pathway Database was used to assess enriched pathways of potential target genes. GO analysis and KEGG pathway analysis were conducted with DAVID Bioinformatics Resources 6.8 (https://david.ncifcrf.gov). Visualization results were achieved using R 3.5.0.

### 2.12 Luciferase reporter assays

The 293T cells (Hanbio Biochemistry, Shanghai, China) were plated in 96‐well plates 24 hours prior to transfection. The pGV306 luciferase reporter plasmids containing pmiR‐REPORT control vector, Luc‐Smad2‐wt vector or Luc‐Smad2‐mu vector and miR‐27a‐5p/miR‐432‐5p mimic or normal control were co‐transfected into 293T cells, using 0.8mg/ml transfection reagent (Hanbio Biochemistry). The luciferase assay was carried out following the instructions of Promega Dual‐Luciferase system. The assay was performed at least 3 times in independent experiments.

### 2.13 Statistical analysis

All analyses were performed using Statistical Package for Social Sciences (SPSS) software (Version 20.0, Inc). Results were expressed as mean ± SEM. Multi‐group comparisons of the means were carried out by one‐way analysis of variance (ANOVA) test with post hoc contrasts by Student‐Newman‐Keuls test. A *P*‐value < .05 was considered to indicate statistical significance.

## 3 RESULTS

### 3.1 Human skin primary fibroblasts were obtained and interfered with SIRT1 siRNAs

**Figure 1:**
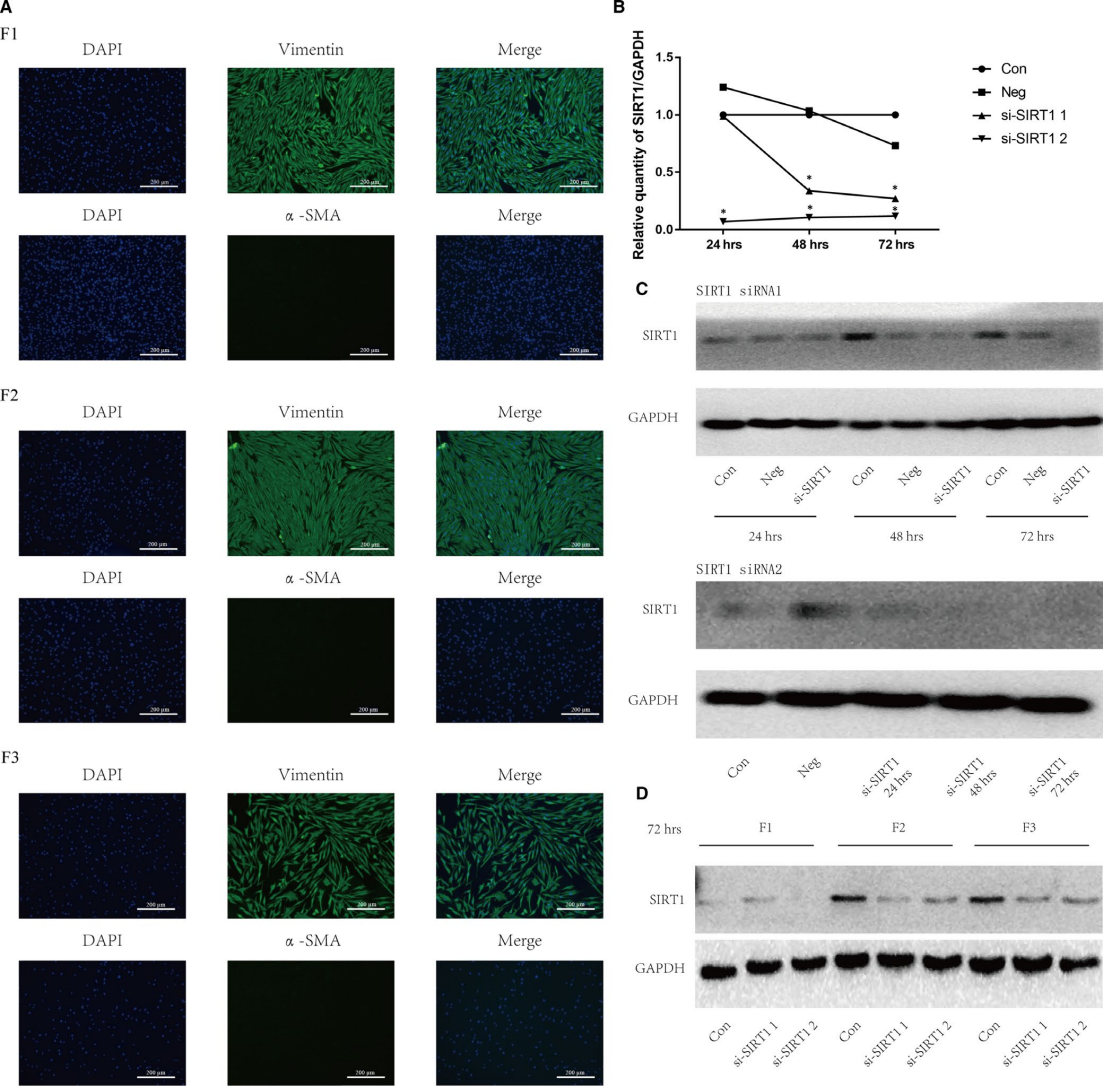
Human skin primary fibroblasts were obtained and interfered with SIRT1 siRNAs. (A) Under inverted fluorescence microscopy, the three skin primary fibroblasts (F1, F2, F3) were identified by vimentin (+) DAPI (+) α‐SMA (‐). (B) The mRNA expressions of SIRT1 in fibroblast cells were tested using qRT‐PCR at transfected times of 24, 48 or 72 hours. All groups were normalized to expression levels via comparing with blank control group. (C) The protein expressions of SIRT1 were tested using Western blot analysis at times of 24, 48 or 72hours, which were transfected with SIRT1 siRNA 1 (up) and SIRT1 siRNA 2 (down). (D) The protein expressions of SIRT1 in three skin primary fibroblasts at 72hours

Cells obtained from foreskin tissues were identified as human skin fibroblasts by results of vimentin (+) DAPI (+) α‐SMA (‐) using immunofluorescence staining (Figure 1A).

Two different SIRT1 siRNAs (siRNA 1 and siRNA 2) were, respectively, transfected into cells, and the transfected efficiencies were verified by qPCR (Figure 1B) and Western blot (Figure 1C). The siRNA 2 showed better transfection efficiencies than siRNA 1 from 24 hours to 72 hours (Figure 1B,C). Thus, siRNA 2 and 72 hours were used for the subsequent investigations. Western blot was used to verify the successful knockdown of SIRT1 in 3 samples at 72hours (Figure 1D).

### 3.2 UVA caused MMP1 increase and SIRT1 decrease in skin primary fibroblasts

**Figure 2:**
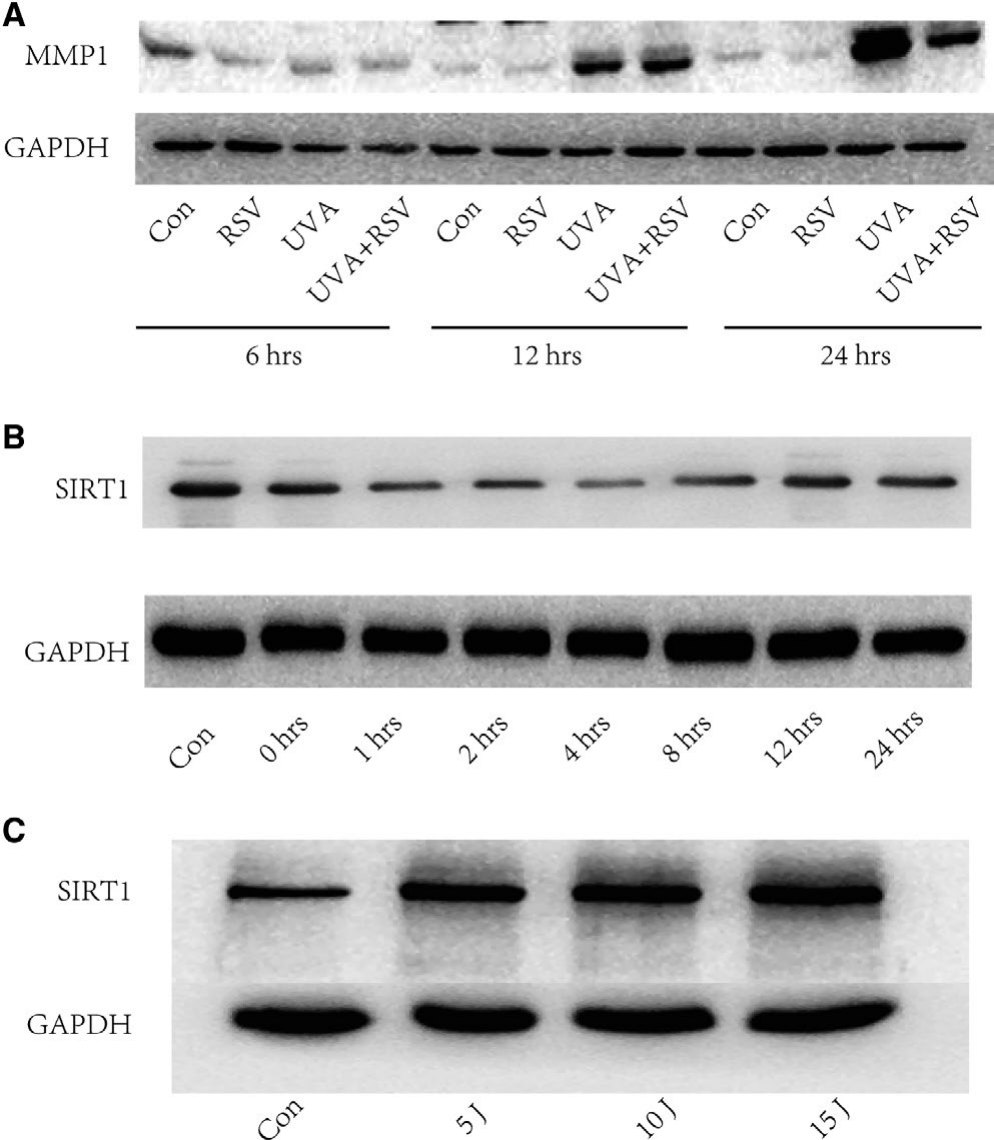
UVA caused MMP1 increase and SIRT1 decrease in skin primary fibroblasts tested by Western blot analysis. (A) The protein expressions of MMP1 in skin primary fibroblasts of different groups. (B) The protein expressions of SIRT1 in skin primary fibroblasts at series time points. (C) The expressions protein of SIRT1 in UVA‐induced fibroblast cells at 24 hours. Note: Con, fibroblasts without any disposal; RSV, Resveratrol

Our group has previously reported that 5 J/cm^2^ of UVA radiation could significantly increase the mRNA expression of MMP1 in skin fibroblasts, and MMP1 presented sinusoidal increase from 6 hours to 48 hours (peaking at 24 hours). In addition, the mRNA level of MMP1 was inhibited from 6hours to 48 hours when treated with 0.01 mmol/L RSV.^20^ The present study validated the previous data by showing UVA radiation (5 J/cm^2^) could induce the protein levels of MMP1 at 12 hours compared with UVA‐free group. In addition, increased expression of MMP1 could be decreased by 0.01 mmol/L RSV at 24 hours. (Figure 2A).

When we detected the protein level of SIRT1 at different time points, we observed that SIRT1 was inhibited as soon as 1 hr after UVA radiation, and restored 8 hours later (Figure 2B). We have also found a UVA dose‐dependent increase in SIRT1 expression at 24 hours (Figure 2C) western‐blotWestern‐blot.

### 3.3 Inhibition of SIRT1 increased the expression level of miR‐27a‐5p

**Figure 3:**
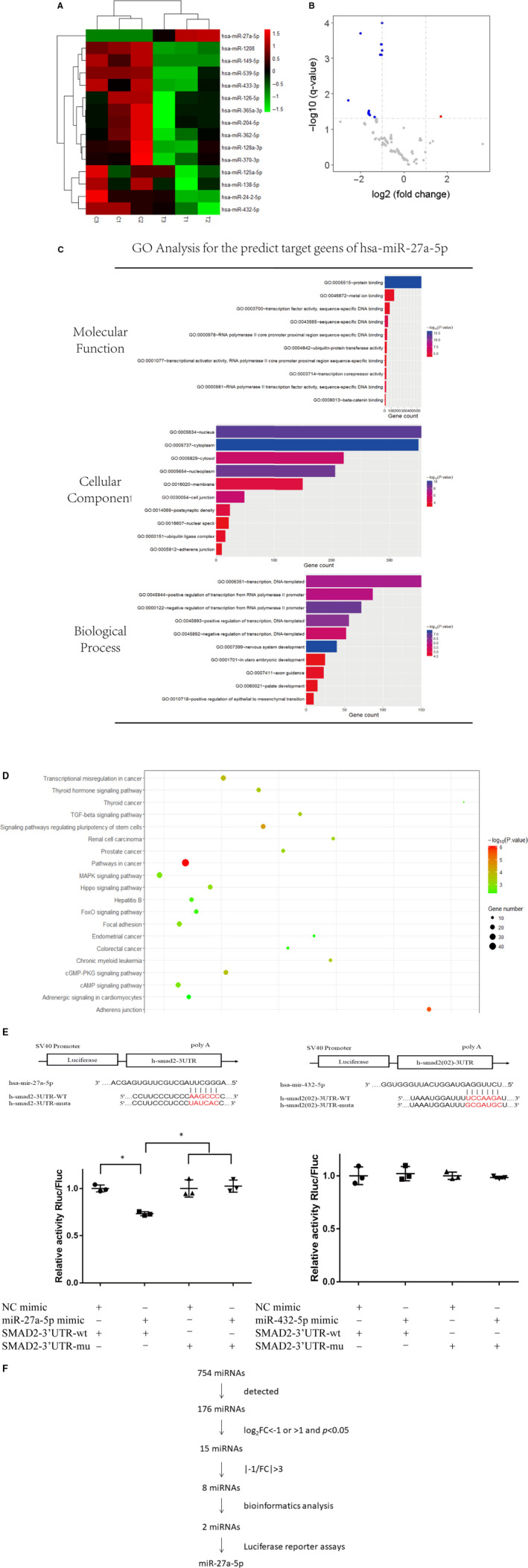
Inhibition of SIRT1 led to the increased expression level of miR‐27a‐5p. (A) Heatmap of differentially expressed microRNAs between si‐SIRT1 cells and normal cells. Note: (C), skin primary fibroblasts without any disposal; T, skin primary fibroblasts transfected with SIRT1 siRNA. (B) Volcano plot of differentially expressed microRNAs between si‐SIRT1 cells and normal cells. (C) GO analysis for the predict target genes of has‐miR‐27a‐5p. (D) Bubble chart of KEGG pathway analysis for the predict target genes of has‐miR‐27a‐5p. (E) Predict binding positions of miR‐27a‐5p/432‐5p on the 3’‐UTR of SMAD2 by TargetScan, and the expression of a luciferase reporter containing SMAD2 3’‐UTR was repressed by miR‐27a‐5p mimic but not by miR‐432‐5p. Asterisk (*) represents statistical significance (*P* < .05) (F) Screening process of differentially expressed microRNAs

Many studies have reported the close associations between miRNAs and SIRT1.^18^, ^19^ In the present study, after successfully silencing SIRT1, a total of 176 miRNAs were detectable in all groups by the microarray. Among of these miRNAs, 15 miRNAs were identified to be differentially expressed between si‐SIRT1 cells and normal cells (log_2_fold change (FC)<−1 or > 1 and *P* < .05). The heatmap analysis and volcano plot analysis displayed that 14 miRNAs were decreased and 1 miRNA (miR‐27a‐5p) was increased in si‐SIRT1–treated cells (Figure 3A,B). The details of differentially expressed microRNAs were listed in Table 1.

**Table 1:** The meaningful differentially expressed microRNAs

	*P* value	log_10_ *p*	Ave FC	log_2_ FC	−1/FC
hsa‐miR‐125a‐5p	.0001	−4	0.494165564	−1.016933616	−2.02361
hsa‐miR‐362‐5p	.0002	−3.698970004	0.248057519	−2.011253407	**−4.03132**
hsa‐miR‐128a‐3p	.0004	−3.397940009	0.485516491	−1.042407795	−2.05966
hsa‐miR‐24‐2‐5p	.0004	−3.397940009	0.48660356	−1.03918122	−2.05506
hsa‐miR‐138‐5p	.0006	−3.22184875	0.497431914	−1.007429024	−2.01033
hsa‐miR‐126‐5p	.0008	−3.096910013	0.486092363	−1.040697626	−2.05722
hsa‐miR‐1208	.0008	−3.096910013	0.46555449	−1.102978061	−2.14798
hsa‐miR‐149‐5p	.0155	−1.809668302	0.167813961	−2.575065354	**−5.95898**
hsa‐miR‐365a‐3p	.0305	−1.515700161	0.325150892	−1.620818712	**−3.0755**
hsa‐miR‐432‐5p	.0331	−1.480172006	0.319899451	−1.644309579	**−3.12598**
hsa‐miR‐539‐5p	.0348	−1.458420756	0.327459315	−1.610612423	**−3.05381**
hsa‐miR‐204‐5p	.0362	−1.441291429	0.323470404	−1.628294378	**−3.09147**
hsa‐miR‐370‐3p	.0389	−1.410050399	0.330941377	−1.595352416	**−3.02168**
hsa‐miR‐27a‐5p	.0441	−1.355561411	3.261247068	1.705423742	**3.3**
hsa‐miR‐433‐3p	.0445	−1.351639989	0.392632298	−1.348749242	−2.54691

Note

Bold values means values with expression changes of more than 3 times compared with the control group, which are used for further screening of differential miRNAs.

In the process of data mining, we chose a FC of 3 as the cut‐off. We ultimately identified 7 miRNAs (miR‐362‐5p, miR‐149‐5p, miR‐365a‐3p, miR‐432‐5p, miR‐539‐5p, miR‐204‐5p and miR‐370‐3p) were significantly down‐regulated, and 1 miRNA of miR‐27a‐5p was significantly up‐regulated in SIRT1 silenced cells compared to control cells.

When predicted target genes of the 8 miRNAs were analysed using GO and KEGG pathway analysis, only miR‐27a‐5p and miR‐432‐5p got positive results. GO analysis showed that the predicted target genes of the two miRNAs mostly enriched in nucleus, acted as molecular binding and involved in transcription process. KEGG pathway analysis revealed that the predicated target genes of miR‐27a‐5p, however miR‐432‐5p not, were enriched in TGF‐beta signalling pathway and MAPK signalling pathway which were closely associated with cell ageing.^21^, ^22^ The GO and KEGG analyses of predicted target genes of miR‐27a‐5p were shown in Figure 3C and Figure 3D, respectively.

Among the predicated target genes of both miR‐27a‐5p and miR‐432‐5p, SMAD2 was the known transcription factor of TGF‐beta signalling pathway.^23^ The luciferase reporting experiment was done to verify the correlation of miRNA and SMAD2, and positive result was found for miR‐27a‐5p and SMAD2, but negative for miR‐432‐5p and SMAD2 (Figure 3E). So, miR‐27a‐5p was chosen for the subsequent experiments.

The entire screening process was shown in Figure 3F. Furthermore, the characters of GO analysis (top 10) and KEGG pathway analysis (top 10) of the predicted target genes of miR‐27a‐5p were shown in Table 2. The predicted target genes of miR‐27a‐5p were presented in Table S2.

**Table 2:** The characters of GO analysis (top 10) and KEGG pathway (top 10) of the predict target genes of miR‐27a‐5p

Category	Term	Count	%	*P* value	Fold enrichment	Bonferroni	Benjamini	FDR
GOTERM_MF_DIRECT	GO:0005515~protein binding	564	58.68887	7.79E‐14	1.232953	7.70E‐11	7.70E‐11	1.23E‐10
GOTERM_MF_DIRECT	GO:0043565~sequence‐specific DNA binding	54	5.619147	1.98E‐06	2.002043	0.001958	9.79E‐04	0.003123
GOTERM_MF_DIRECT	GO:0000978~RNA polymerase II core promoter proximal region sequence‐specific DNA binding	39	4.058273	2.02E‐05	2.109821	0.019793	0.006642	0.031859
GOTERM_MF_DIRECT	GO:0000981~RNA polymerase II transcription factor activity, sequence‐specific DNA binding	24	2.497399	2.72E‐05	2.695407	0.026567	0.006709	0.042908
GOTERM_MF_DIRECT	GO:0003714~transcription corepressor activity	26	2.705515	5.57E‐05	2.459725	0.053634	0.010965	0.087825
GOTERM_MF_DIRECT	GO:0046872~metal ion binding	146	15.19251	8.70E‐05	1.355195	0.082555	0.014258	0.13724
GOTERM_MF_DIRECT	GO:0003700~transcription factor activity, sequence‐specific DNA binding	78	8.116545	9.59E‐05	1.558765	0.090542	0.013467	0.151156
GOTERM_MF_DIRECT	GO:0004842~ubiquitin‐protein transferase activity	34	3.537981	2.43E‐04	1.984688	0.2135	0.029574	0.382059
GOTERM_MF_DIRECT	GO:0001077~transcriptional activator activity, RNA polymerase II core promoter proximal region sequence‐specific binding	27	2.809573	2.52E‐04	2.197157	0.220502	0.027299	0.396259
GOTERM_MF_DIRECT	GO:0008013~beta‐catenin binding	14	1.456816	2.97E‐04	3.278865	0.254483	0.028941	0.466993
GOTERM_BP_DIRECT	GO:0007399~nervous system development	40	4.162331	3.70E‐08	2.677744	1.32E‐04	1.32E‐04	6.79E‐05
GOTERM_BP_DIRECT	GO:0000122~negative regulation of transcription from RNA polymerase II promoter	72	7.492196	1.45E‐07	1.921281	5.17E‐04	2.59E‐04	2.66E‐04
GOTERM_BP_DIRECT	GO:0045893~positive regulation of transcription, DNA‐templated	56	5.827263	3.14E‐07	2.08916	0.00112	3.73E‐04	5.77E‐04
GOTERM_BP_DIRECT	GO:0006351~transcription, DNA‐templated	150	15.60874	7.07E‐07	1.474129	0.002519	6.30E‐04	0.001298
GOTERM_BP_DIRECT	GO:0045944~positive regulation of transcription from RNA polymerase II promoter	87	9.05307	1.13E‐06	1.703889	0.004019	8.05E‐04	0.002073
GOTERM_BP_DIRECT	GO:0045892~negative regulation of transcription, DNA‐templated	52	5.41103	3.05E‐06	2.002137	0.010832	0.001814	0.005605
GOTERM_BP_DIRECT	GO:0007411~axon guidance	23	2.39334	2.58E‐05	2.779212	0.08777	0.013038	0.047269
GOTERM_BP_DIRECT	GO:0060021~palate development	15	1.560874	3.21E‐05	3.792003	0.108146	0.014205	0.058888
GOTERM_BP_DIRECT	GO:0010718~positive regulation of epithelial to mesenchymal transition	10	1.040583	3.32E‐05	5.822065	0.111528	0.013053	0.060843
GOTERM_BP_DIRECT	GO:0001701~in utero embryonic development	25	2.601457	3.97E‐05	2.568558	0.131928	0.014048	0.07279
GOTERM_CC_DIRECT	GO:0005737~cytoplasm	350	36.4204	4.70E‐11	1.343727	2.68E‐08	2.68E‐08	6.90E‐08
GOTERM_CC_DIRECT	GO:0005634~nucleus	355	36.94069	5.51E‐10	1.314346	3.14E‐07	1.57E‐07	8.08E‐07
GOTERM_CC_DIRECT	GO:0005654~nucleoplasm	206	21.436	1.69E‐09	1.483467	9.66E‐07	3.22E‐07	2.49E‐06
GOTERM_CC_DIRECT	GO:0030054~cell junction	49	5.098855	1.02E‐06	2.140244	5.79E‐04	1.45E‐04	0.001489
GOTERM_CC_DIRECT	GO:0005829~cytosol	221	22.99688	1.79E‐06	1.33656	0.00102	2.04E‐04	0.002627
GOTERM_CC_DIRECT	GO:0014069~postsynaptic density	24	2.497399	4.56E‐05	2.615009	0.025638	0.004319	0.066801
GOTERM_CC_DIRECT	GO:0016020~membrane	150	15.60874	4.67E‐05	1.366937	0.026254	0.003793	0.068428
GOTERM_CC_DIRECT	GO:0000151~ubiquitin ligase complex	16	1.664932	2.95E‐04	2.970134	0.155	0.020832	0.432387
GOTERM_CC_DIRECT	GO:0005912~adherens junction	10	1.040583	7.21E‐04	4.009681	0.337216	0.044672	1.052669
GOTERM_CC_DIRECT	GO:0016607~nuclear speck	22	2.289282	0.001104	2.194353	0.467317	0.06104	1.607427
KEGG_PATHWAY	hsa05200:Pathways in cancer	45	4.682622	8.96E‐07	2.187977	2.23E‐04	2.23E‐04	0.001163
KEGG_PATHWAY	hsa04520:Adherens junction	16	1.664932	2.72E‐06	4.306103	6.78E‐04	3.39E‐04	0.003534
KEGG_PATHWAY	hsa04550:Signaling pathways regulating pluripotency of stem cells	21	2.185224	3.42E‐05	2.86625	0.008476	0.002833	0.044373
KEGG_PATHWAY	hsa05202:Transcriptional misregulation in cancer	22	2.289282	1.46E‐04	2.517265	0.035702	0.009047	0.189372
KEGG_PATHWAY	hsa04022:cGMP‐PKG signalling pathway	21	2.185224	1.91E‐04	2.539715	0.046557	0.00949	0.248268
KEGG_PATHWAY	hsa04919:Thyroid hormone signalling pathway	17	1.768991	2.84E‐04	2.82471	0.068188	0.011702	0.367553
KEGG_PATHWAY	hsa05220:Chronic myeloid leukaemia	13	1.352758	3.04E‐04	3.450116	0.073019	0.010773	0.394552
KEGG_PATHWAY	hsa04350:TGF‐beta signalling pathway	14	1.456816	3.67E‐04	3.184722	0.087399	0.011367	0.475717
KEGG_PATHWAY	hsa05211:Renal cell carcinoma	12	1.248699	5.40E‐04	3.474242	0.125873	0.014837	0.69898
KEGG_PATHWAY	hsa05215:Prostate cancer	14	1.456816	5.83E‐04	3.039962	0.135196	0.01442	0.754482

### 3.4 SIRT1 up‐regulated SMAD2 via down‐regulating miR‐27a‐5p

**Figure 4:**
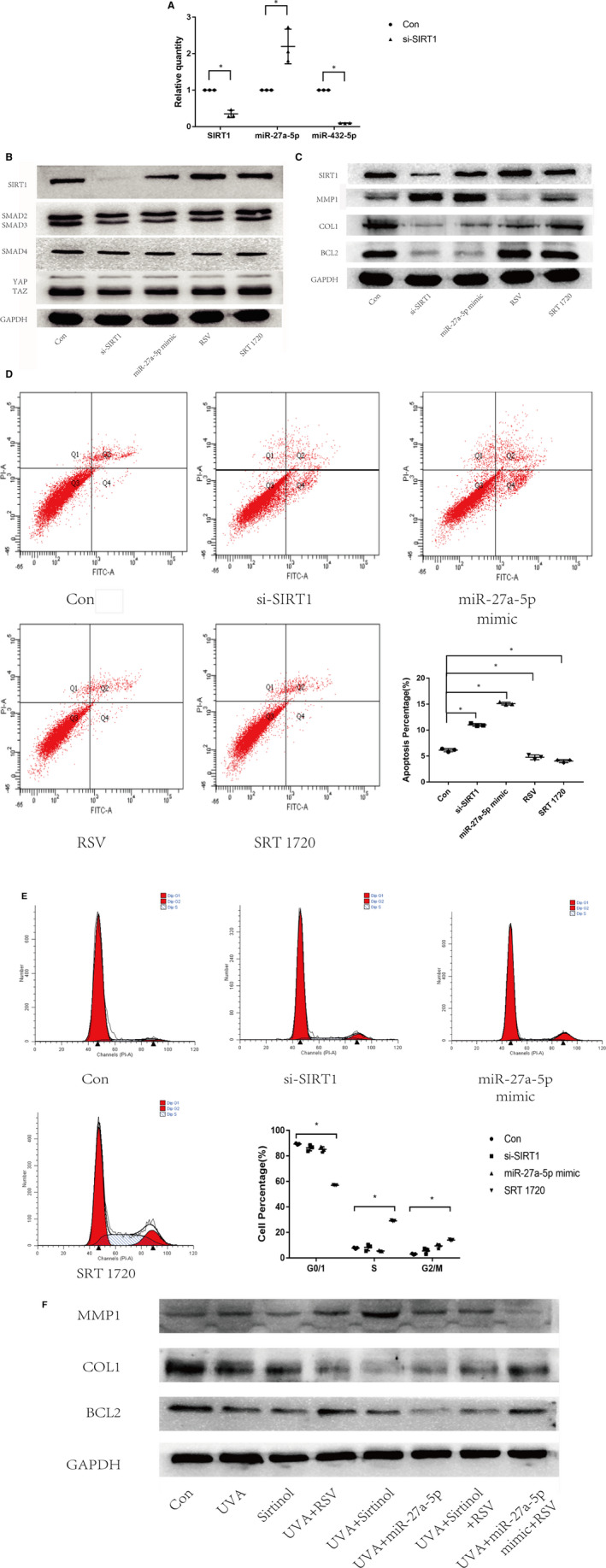
UVA affected the SIRT1‐SMAD2‐miR‐27a‐5p‐MMP1/COL1/BCL2 signalling axis. (A) Relative quantities of SIRT1, miR‐27a‐5p, miR‐432‐5p tested by qRT‐PCR assay. (B) Protein expressions of SIRT1, SMAD2/3, SMAD4 and YAP/TAZ in fibroblasts tested by Western blot analysis. (C) Protein expressions of SIRT1, MMP1, COL1 and BCL2 in fibroblasts tested by Western blot analysis. (D) The distributions of apoptotic and necrotic fibroblast cells were assessed with flow cytometry. The apoptotic cells were significantly more in si‐SIRT1 group than in control group (*P* < .05). Horizontal coordinates indicate Annexin V staining, and vertical coordinates indicate PI staining. (E) The distributions of cell cycle were assessed with flow cytometry. Horizontal coordinates indicate PI staining,, and vertical coordinates indicate number of cells. (F) Protein expressions of SIRT1, MMP1, COL1 and BCL2 in UVA‐induced fibroblasts tested by Western blot analysis. Note: Con, fibroblast cells without any disposal; si‐SIRT1, fibroblast cells transfected with SIRT1 siRNA. RSV, Resveratrol. Asterisk (*) represents statistical significance (*P* < .05) compared to control group

Having found SIRT1 negatively regulated miR‐27a‐5p by miRNA microarrays, we then validated these results by qRT‐PCR assay (Figure 4A). According to the facts that SIRT1 down‐regulated miR‐27a‐5p and miR‐27a‐5p has the combined position on the 3'‐UTR of SMAD2, we assumed that knockdown of SIRT1 could decrease SMADs through the increased miR‐27a‐5p. So we tested the protein levels of SMAD2 and association proteins including SMAD3/4, YAP and TAZ by Western blot, under different conditions. SMAD2 was obviously decreased by SIRT1 siRNA or by miR‐27a‐5p mimic (*P* < .05), although uninfluenced by RSV or SRT 1720. The expression patterns of SMAD3 and TAZ were similar to those of SMAD2 (Figure 4B). No significant differences were seen for SMAD4 or YAP among groups. These results revealed SIRT1 could regulate SMAD2 via miR‐27a‐5p.

### 3.5 SIRT1 down‐regulated MMP1 and up‐regulated COL1 and BCL2 via miR‐27a‐5p

We demonstrated SIRT1 could regulate SMAD2 via miR‐27a‐5p. Previous studies supported that SMAD2 could regulate MMP1, COL1 ^24^and BCL2,^25^ and the latter markers were closely related to ageing and cell apoptosis. So we speculated SIRT1 could regulate MMP1, COL1 and BCL2. The results showed that absence of SIRT1 led to increased expression of MMP1 and decreased expression of COL1 and BCL2. When miR‐27a‐5p was overexpressed by mimic, we detected the similar changes in these downstream targets. In addition, compared to untreated cells, both RSV and SRT 1720 successfully up**‐**regulated SIRT1, decreased MMP1, as well as increased COL1 and BCL2 (Figure 4C). These results revealed a signalling axis of SIRT1‐miR‐27a‐5p‐SMAD2‐MMP1/COL1/BCL2.

### 3.6 SIRT1 decreased cell apoptosis and induces cell arrest in G2/M phase via miR‐27a‐5p

In the further investigation, we used flow cytometry assay to observe the impact of SIRT1 on cell apoptosis and cell cycle in skin primary fibroblasts. Apoptosis analysis showed that silencing of SIRT1 by siRNA strategy significantly increased the percentage of apoptotic cells (*P* < .05), which was significantly decreased when SIRT1 was up**‐**regulated by RSV and SRT 1720 (*P* < .05). The percentage of apoptotic cells was also significantly increased in miR‐27a‐5p mimic**‐**treated cells (*P* < .05), consistent with that in the si‐SIRT1 group (Figure 4D). These data displayed that SIRT1 could regulate cell apoptosis via miR‐27a‐5p. Cell cycle analysis showed activating SIRT1 by SRT 1720 decreased the percentage of G0/1 phase cells and increased the percentage of S and G2/M phase cells. When skin primary fibroblasts were pre‐treated with SIRT1 siRNA or miR‐27a‐5p mimic, the percentage of G0/1 phase cells was decreased and the percentages of S and G2/M phase cells were increased (Figure 4E).

### 3.7 UVA affected the SIRT1‐miR‐27a‐5p‐MMP1/COL1/BCL2 axis

Being the downstream targets of SIRT1, MMP1, COL1 and BCL2 were also involved in the process of UVA‐induced cellular damages. We hypothesized that UVA may affect MMP1, COL1 and BCL2 via SIRT1. We initially treated the skin primary fibroblasts with UVA radiation or 10 uM Sirtinol. After 24 hours of treatment, we see that UVA or Sirtinol demonstrated contrary impacts on MMP1, COL1 or BCL2 against SIRT1 (Figure 4F).

Since UVA radiation and Sirtinol both caused increased MMP1 and decreased COL1 and BCL2, it was not surprise to see additive impact on these three target proteins under the combination of UVA radiation and Sirtinol. In addition, when RSV was given to UVA and Sirtinol pre‐treated skin primary fibroblasts, reversed changes in MMP1, COL1 and BCL2 were detected (Figure 4F). These results further confirmed that UVA could regulate MMP1, COL1 and BCL2 via SIRT1 in skin primary fibroblasts.

As we have found that SIRT1 regulated MMP1, COL1 and BCL2 via miR‐27a‐5p, we came to the question that if miR‐27a‐5p was also involved in the UVA‐SIRT1‐MMP1/COL1/BCL2 axis. To address this question, skin primary fibroblasts were dealt with UVA radiation and miR‐27a‐5p was found to be up**‐**regulated. Under the treatment of miR‐27a‐5p mimic, the protein levels of MMP1, COL1 and BCL2 were consistently altered with that of SIRT1 knocking down. Additional treatment of RSV has reversed the impact of UVA + miR‐27a‐5p mimic on three target proteins by showing decreased MMP1 and increased COL1 and BCL2. All these results suggested the UVA‐SIRT1‐ miR‐27a‐5p‐MMP1/COL1/BCL2 axis (Figure 4F).

**Figure 5:**
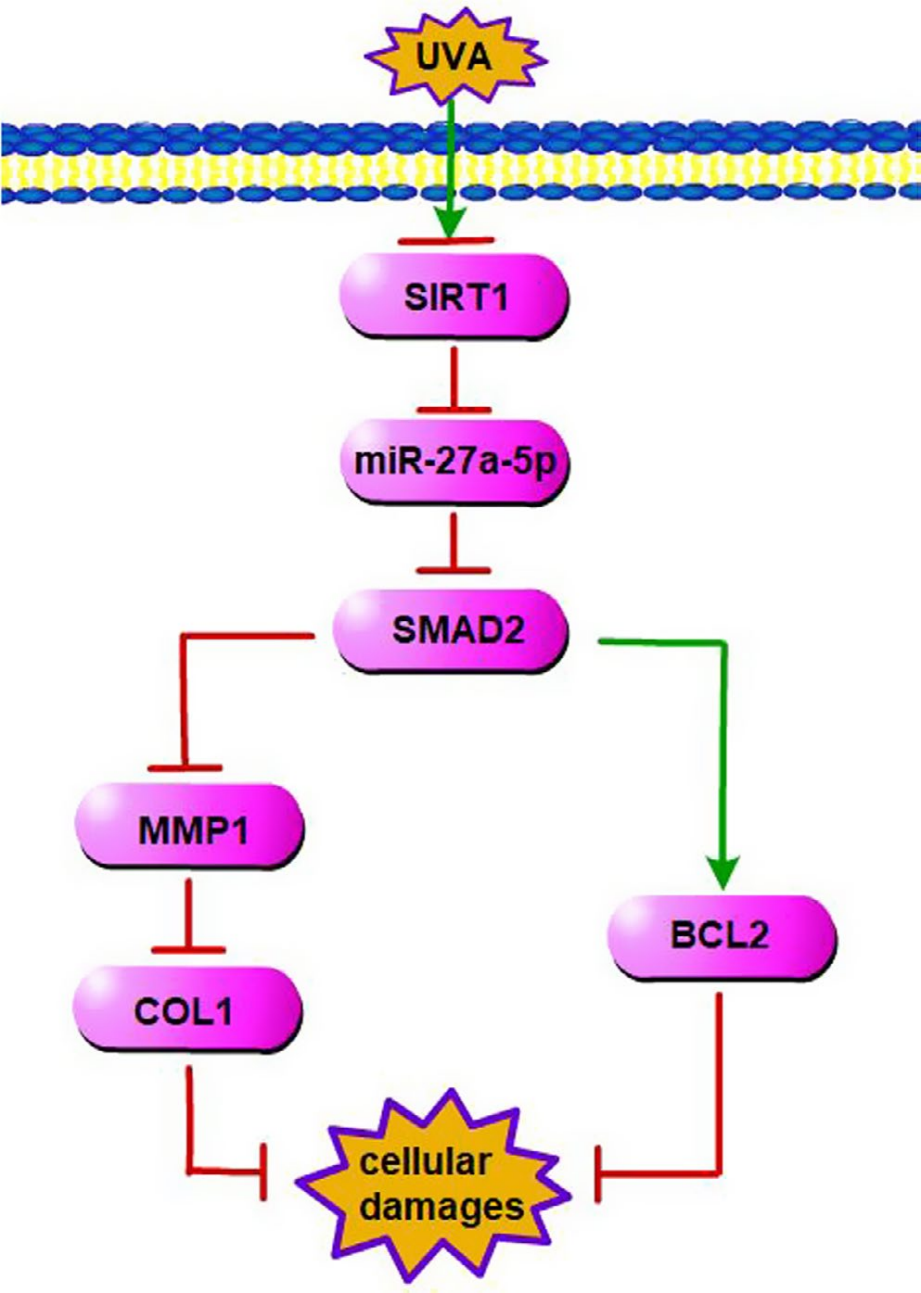
Schematic illustration of this study

The schematic illustration of this study was shown in Figure 5. UVA down**‐**regulated SIRT1 and SIRT1 down**‐**regulated miR‐27a‐5p, then regulated the damages of skin primary fibroblasts through influencing SMAD2‐MMP1/COL1/BCL2 axis.

## 4 DISCUSSION

We have initially found that the SIRT1 protein level showed a decrease‐restore feedback pattern after UVA radiation, suggesting SIRT1 participated in the process of autonomous cell repair in fibroblast cells over time. The transient decreasing of SIRT1 after UVA radiation may contribute to the overexpression of MMP1. As the downstream target of SIRT1, MMP1 presented a time delay in protein translation compared to SIRT1. Since increased expression of MMP1 could be decreased again by resveratrol, an activator of SIRT1, we could further conclude that SIRT1 negatively regulated MMP1.

Many studies have reported that miRNAs including miR‐30a, miR‐132, miR‐212, miR‐221 or miR‐4262 regulated SIRT1 to participate in various biological processes in different cells.^18^, ^19^, ^26^, ^27^ However, our study identified downstream miRNAs of SIRT1 that could be positively or negatively regulated by SIRT1 using PCR microarray. We ultimately chose miR‐27a‐5p as the target miRNA after a series of bioinformatics analysis. As far as we know, this is the first study revealing miR‐27a‐5p was a downstream effector of SIRT1. As the regulator of SIRT1, UVA was also identified to affect miR‐27a‐5p. In addition to the miR‐27a‐5p in our study, UVA has also been reported to regulate other miRNAs such as miR‐101, miR‐19 and miR‐206 in various human cell lines. Upon activation by UVA, these miRNAs exerted their functions in mediating biological processes such as photoageing, cell survival or photocarcinogenesis.^28^, ^29^, ^30^

UVA‐induced increased ROS could up**‐**regulate MMP1 via MAPKs pathway, which in turn led to degradation of collagen.^31^ In addition, BCL2 was detected to be a target gene of miR‐27a‐5p by target gene prediction analysis. Therefore, we went to determine whether MMP1, COL1 or BCL2 was downstream effectors of UVA‐SIRT1‐miR‐27a‐5p axis. Regulations of these three targets by UVA‐SIRT1‐miR‐27a‐5p were confirmed by inhibiting and/or activating SIRT1, or overexpressing miR‐27a‐5p. As UVA radiation, SIRT1 silencing or miR‐27a‐5p mimic all consistently caused increased MMP1 and decreased COL1/BCL2, it was not surprising to observe enhanced changes in UVA radiation + SIRT1 inhibitors/miR‐27a‐5p mimic**‐**treated cells. Moreover, these changes could be reversed when SIRT1 agonists were given. All these data have supported that MMP1, COL1 and BCL2 were downstream target genes for UVA‐SIRT1‐miR‐27a‐5p axis.

SIRT1 has been previously reported to protect cells against apoptosis. Activated SIRT1 inhibited Mn‐induced apoptosis and FOXO3a activation, thus, impeded Mn‐induced neurotoxicity.^32^ In psoriatic fibroblasts, oxidative stress, mitochondrial damage and apoptosis, and reduced SIRT1 expression were simultaneously observed.^33^ Our study also found the protective role of SIRT1 by showing decreased apoptotic cells when SIRT1 was overexpressed by RSV/SRT 1720, or increased apoptotic cells when SIRT1 was knocked down in human fibroblast cells.

The protective role of SIRT1 against apoptosis was likely to be executed via down**‐**regulating miR‐27a‐5p, as shown by increasing of apoptotic cells when the cells were treated with miR‐27a‐5p mimic. Some other miRNAs such as miR‐3613‐3p ^34^ and miR‐125b ^35^ have also been reported to inhibit apoptosis in BE(2)C cells and myeloma cells, respectively. In contrary, certain miRNAs played roles in inducing apoptosis. For example, apoptosis was promoted by miR‐99a in human granulosa cells ^36^ and miR‐223 in hepatic carcinoma cells.^37^

In the analysis of cell cycle, we observed significant decreased percentage of cells in G0/G1 phase, and increased percentage of cells in G2/M phase by SRT 1720, suggesting SIRT1 induced cell cycle arrest in G2/M phase. In consistency with our finding, Cao et al found the activation of SIRT1 could rescue the G0/G1 phase cell cycle arrest in TM4SF1‐deficient BCa cells.^38^

However, other interference factors such as SIRT1 siRNA or miR‐27a‐5p mimic failed to cause significant changes in fibroblasts. We speculated the reason was that the original expression level of SIRT1 was not enough to induce cell cycle changes, and therefore, we could not detect cell cycle changes when down**‐**regulated SIRT1 or up**‐**regulated miR‐27a‐5p. The exact regulatory function of SIRT1 or miR‐27a‐5p on cell cycle should be achieved on more primary fibroblasts in future study.

In conclusion, the present study identified a novel SIRT1‐miR‐27a‐5p‐SMAD2‐MMMP1/COL1/BCL2 axis and a mechanism of UVA influencing human primary fibroblasts through this axis. Our finding may provide potential therapeutic targets for UVA‐induced skin damage. We suspected that increasing the expression of SIRT1 or decreasing the expression of miR‐27a‐5p may reduce UVA‐related diseases, or even play a role in the treatment of UVA‐induced skin cancer. However, limitations, such as the unclear relationships between MMP1 and BCL2, and the lack of animal work, should be addressed in future studies.

## CONFLICT OF INTEREST

The authors confirm that there are no conflicts of interest.

## AUTHOR CONTRIBUTION

**Shibin Jiang:** Data curation (lead); Methodology (lead); Writing‐original draft (lead); Writing‐review & editing (lead). **Yansong Lu:** Methodology (supporting). **Tao Liu:** Resources (lead). **Liangman Li:** Validation (equal). **Hexiao Wang:** Conceptualization (equal); Writing‐original draft (supporting). **Yan Wu:** Conceptualization (equal); Writing‐original draft (supporting); Writing‐review & editing (supporting). **Xing‐Hua Gao:** Validation (equal). **Hong‐Duo Chen:** Validation (equal). 

## Supporting information

Fig S1

Table S1

Table S2

## Data Availability

No data sets were generated for this study.
