# Corrigendum

**DOI:** 10.1111/jcmm.17172

**Published:** 2022-02-05

In Shi‐Bin Jiang et al.^1^, the band of GAPDH in Figure 4C was duplicated in Figure 4B. The correct image is shown below. The authors confirm all results and conclusions of this article remain unchanged.

**FIGURE 4:**
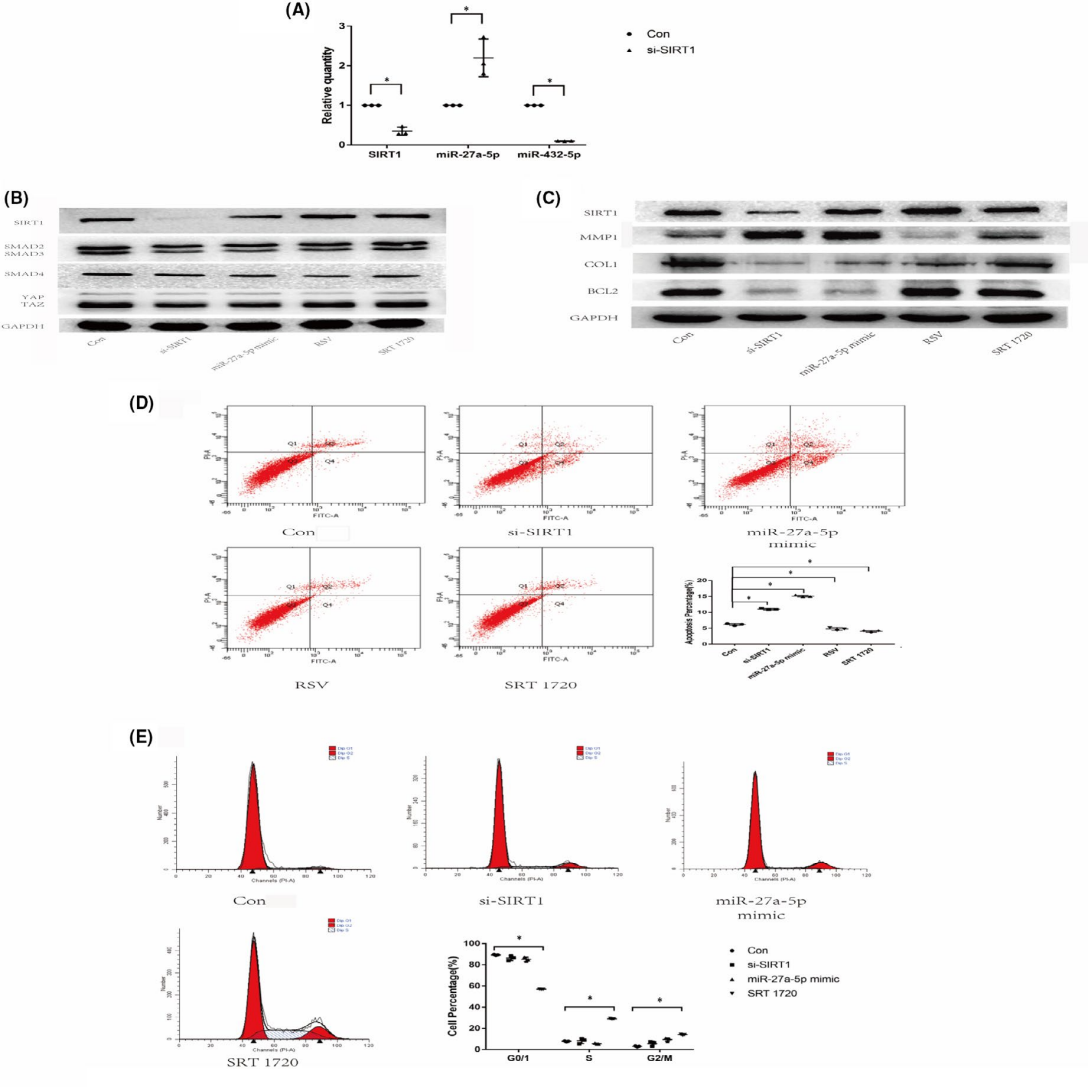
UVA affected the SIRT1‐SMAD2‐miR‐27a‐5p‐MMP1/COL1/BCL2 signalling axis. A, Relative quantities of SIRT1, miR‐27a‐5p, miR‐432‐5p tested by qRT‐PCR assay. B, Protein expressions of SIRT1, SMAD2/3, SMAD4 and YAP/TAZ in fibroblasts tested by Western blot analysis. C, Protein expressions of SIRT1, MMP1, COL1 and BCL2 in fibroblasts tested by Western blot analysis. D, Distributions of apoptotic and necrotic fibroblast cells were assessed with flow cytometry. The apoptotic cells were significantly more in si‐SIRT1 group than in control group (*P* < .05). Horizontal coordinates indicate Annexin V staining, and vertical coordinates indicate PI staining. E, Distributions of cell cycle were assessed with flow cytometry. Horizontal coordinates indicate PI staining, and vertical coordinates indicate number of cells. F, Protein expressions of SIRT1, MMP1, COL1 and BCL2 in UVA‐induced fibroblasts tested by Western blot analysis. Con, fibroblast cells without any disposal; si‐SIRT1, fibroblast cells transfected with SIRT1 siRNA. RSV, Resveratrol. Asterisk (*) represents statistical significance (*P* < .05) compared with control group
