# Supplementary figures and images for: UVA influenced the SIRT1‐miR‐27a‐5p‐SMAD2‐MMP1/COL1/BCL2 axis in human skin primary fibroblasts
Source: J Cell Mol Med. 2020 Aug 13;24(17):10027–41. doi: 10.1111/jcmm.15610 (PMC7520305; doi:10.1111/jcmm.15610)

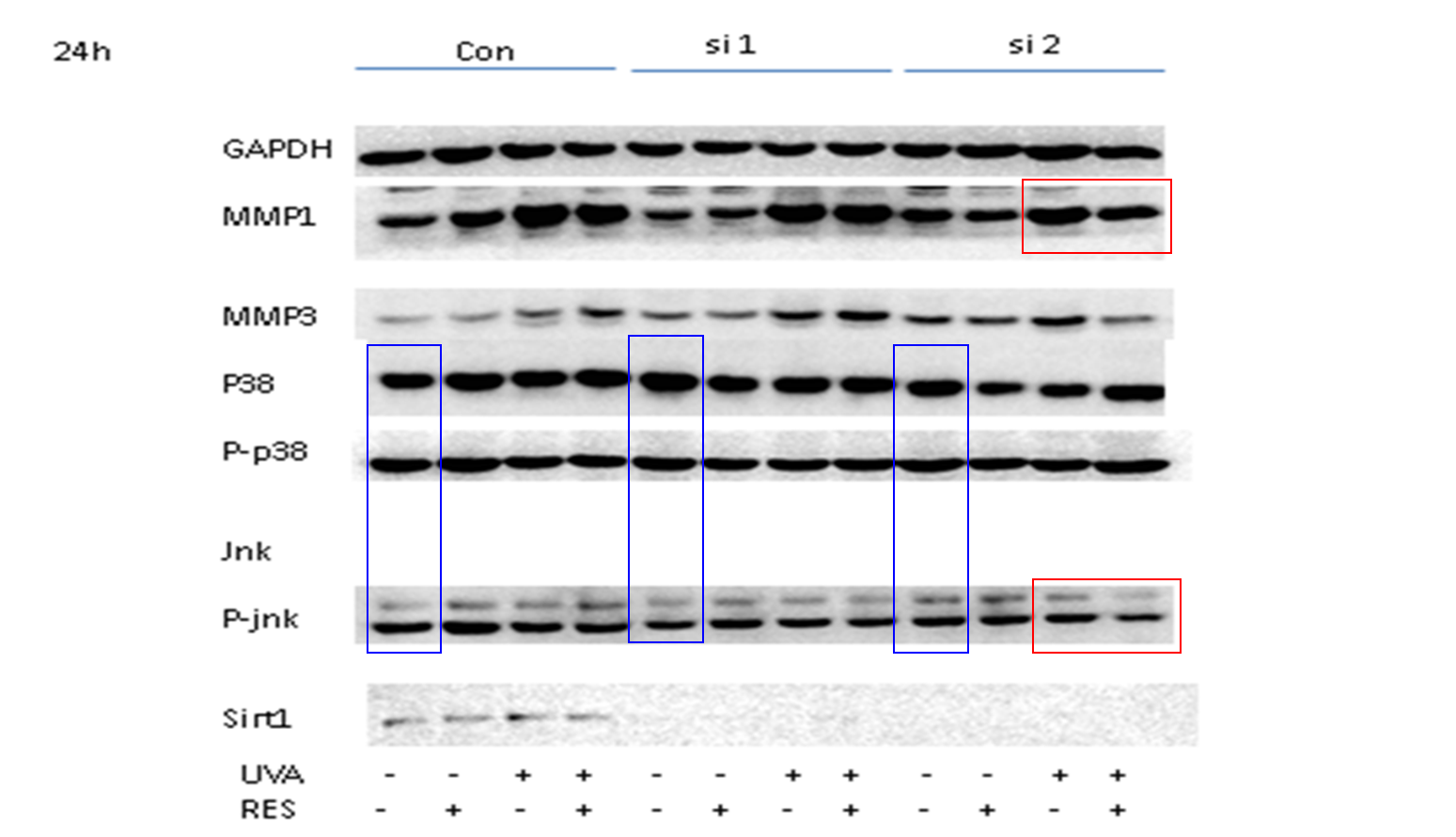

Supplement: Supplementary file 1 — Fig S1 [file JCMM-24-10027-s001.tif]
