# Supplementary material for: UVA influenced the SIRT1‐miR‐27a‐5p‐SMAD2‐MMP1/COL1/BCL2 axis in human skin primary fibroblasts
Source: J Cell Mol Med. 2020 Aug 13;24(17):10027–41. doi: 10.1111/jcmm.15610 (PMC7520305; doi:10.1111/jcmm.15610)
Supplement: Supplementary file 2 — Table S1 [file JCMM-24-10027-s002.docx]

Supplement Table 1 The list of 758 miRNAs in miRNA microarray

| ath-miR159a-000338 | hsa-miR-1224-3P-002752 | hsa-miR-1283-002890 | hsa-miR-145-002278 | hsa-miR-190-000489 | hsa-miR-214-002306 | hsa-miR-29b-000413 | hsa-miR-33a-002135 | hsa-miR-411-001610 | hsa-miR-501-3p-002435 | hsa-miR-520D-3P-002743 | hsa-miR-553-001521 | hsa-miR-601-001558 | hsa-miR-652-002352 | hsa-miR-889-002202 | mmu-miR-615-001960 | |  |
| --- | --- | --- | --- | --- | --- | --- | --- | --- | --- | --- | --- | --- | --- | --- | --- | --- | --- |
| dme-miR-7-000268 | hsa-miR-1225-3P-002766 | hsa-miR-1284-002903 | hsa-miR-146a#-002163 | hsa-miR-190b-002263 | hsa-miR-215-000518 | hsa-miR-29b-1#-002165 | hsa-miR-33b-002085 | hsa-miR-412-001023 | hsa-miR-502-001109 | hsa-miR-520d-5p-002393 | hsa-miR-554-001522 | hsa-miR-603-001566 | hsa-miR-653-002292 | hsa-miR-890-002209 | mmu-miR-93-001090 | |  |
| hsa-let-7a#-002307 | hsa-miR-1226#-002758 | hsa-miR-1285-002822 | hsa-miR-146a-000468 | hsa-miR-191#-002678 | hsa-miR-216a-002220 | hsa-miR-29b-2#-002166 | hsa-miR-340#-002259 | hsa-miR-422a-002297 | hsa-miR-502-3p-002083 | hsa-miR-520e-001119 | hsa-miR-555-001523 | hsa-miR-604-001567 | hsa-miR-654-001611 | hsa-miR-891a-002191 | mmu-miR-96-000186 | |  |
| hsa-let-7a-000377 | hsa-miR-1227-002769 | hsa-miR-1286-002773 | hsa-miR-146b-001097 | hsa-miR-191-002299 | hsa-miR-216b-002326 | hsa-miR-29c-000587 | hsa-miR-340-002258 | hsa-miR-423-5p-002340 | hsa-miR-503-001048 | hsa-miR-520f-001120 | hsa-miR-556-3p-002345 | hsa-miR-605-001568 | hsa-miR-654-3p-002239 | hsa-miR-891b-002210 | rno-miR-29c#-001818 | |  |
| hsa-let-7b#-002404 | hsa-miR-1228#-002763 | hsa-miR-1288-002832 | hsa-miR-146b-3p-002361 | hsa-miR-192#-002272 | hsa-miR-217-002337 | hsa-miR-301-000528 | hsa-miR-342-3p-002260 | hsa-miR-424#-002309 | hsa-miR-504-002084 | hsa-miR-520g-001121 | hsa-miR-556-5p-002344 | hsa-miR-606-001569 | hsa-miR-655-001612 | hsa-miR-892a-002195 | rno-miR-7#-001338 | |  |
| hsa-let-7b-002619 | hsa-miR-1233-002768 | hsa-miR-1289-002871 | hsa-miR-147-000469 | hsa-miR-192-000491 | hsa-miR-218-000521 | hsa-miR-301b-002392 | hsa-miR-342-5p-002147 | hsa-miR-424-000604 | hsa-miR-505#-002087 | hsa-miR-520h-001170 | hsa-miR-557-001525 | hsa-miR-607-001570 | hsa-miR-656-001510 | hsa-miR-892b-002214 | RNU44-001094 | |  |
| hsa-let-7c#-002405 | hsa-miR-1236-002761 | hsa-miR-128a-002216 | hsa-miR-147b-002262 | hsa-miR-193a-3p-002250 | hsa-miR-218-1#-002094 | hsa-miR-302a#-002381 | hsa-miR-345-002186 | hsa-miR-425#-002302 | hsa-miR-505-002089 | hsa-miR-521-001122 | hsa-miR-558-001526 | hsa-miR-608-001571 | hsa-miR-657-001512 | hsa-miR-9#-002231 | RNU48-001006 | |  |
| hsa-let-7c-000379 | hsa-miR-1238-002927 | hsa-miR-129#-002298 | hsa-miR-148a#-002134 | hsa-miR-193a-5p-002281 | hsa-miR-218-2#-002294 | hsa-miR-302a-000529 | hsa-miR-346-000553 | hsa-miR-425-5p-001516 | hsa-miR-506-001050 | hsa-miR-522-002413 | hsa-miR-559-001527 | hsa-miR-609-001573 | hsa-miR-658-001513 | hsa-miR-9-000583 | U6 snRNA-001973 | |  |
| hsa-let-7d-002283 | hsa-miR-124#-002197 | hsa-miR-1290-002863 | hsa-miR-148a-000470 | hsa-miR-193b#-002366 | hsa-miR-219-000522 | hsa-miR-302b#-002119 | hsa-miR-34a#-002316 | hsa-miR-429-001024 | hsa-miR-507-001051 | hsa-miR-523-002386 | hsa-miR-561-001528 | hsa-miR-613-001586 | hsa-miR-659-001514 | hsa-miR-920-002150 |  |  |  |
| hsa-let-7e#-002407 | hsa-miR-1243-002854 | hsa-miR-129-000590 | hsa-miR-148b#-002160 | hsa-miR-193b-002367 | hsa-miR-219-1-3p-002095 | hsa-miR-302b-000531 | hsa-miR-34a-000426 | hsa-miR-431#-002312 | hsa-miR-508-001052 | hsa-miR-524-001173 | hsa-miR-562-001529 | hsa-miR-614-001587 | hsa-miR-660-001515 | hsa-miR-921-002151 |  |  |  |
| hsa-let-7e-002406 | hsa-miR-1244-002791 | hsa-miR-1291-002838 | hsa-miR-148b-000471 | hsa-miR-194#-002379 | hsa-miR-219-2-3p-002390 | hsa-miR-302c#-000534 | hsa-miR-34b-000427 | hsa-miR-431-001979 | hsa-miR-508-5p-002092 | hsa-miR-524-5p-001982 | hsa-miR-563-001530 | hsa-miR-615-5p-002353 | hsa-miR-661-001606 | hsa-miR-922-002152 |  |  |  |
| hsa-let-7f-000382 | hsa-miR-1245-002823 | hsa-miR-1292-002824 | hsa-miR-149#-002164 | hsa-miR-194-000493 | hsa-miR-22#-002301 | hsa-miR-302c-000533 | hsa-miR-34b-002102 | hsa-miR-432#-001027 | hsa-miR-509-3-5p-002155 | hsa-miR-525-001174 | hsa-miR-564-001531 | hsa-miR-616-001589 | hsa-miR-662-001607 | hsa-miR-924-002154 |  |  |  |
| hsa-let-7f-1#-002417 | hsa-miR-1247-002893 | hsa-miR-1293-002905 | hsa-miR-149-002255 | hsa-miR-195#-002107 | hsa-miR-220-000523 | hsa-miR-302d#-002120 | hsa-miR-34c-000428 | hsa-miR-432-001026 | hsa-miR-509-5p-002235 | hsa-miR-525-3p-002385 | hsa-miR-566-001533 | hsa-miR-616-002414 | hsa-miR-663B-002857 | hsa-miR-92a-000431 |  |  |  |
| hsa-let-7f-2#-002418 | hsa-miR-1248-002870 | hsa-miR-1294-002785 | hsa-miR-150-000473 | hsa-miR-195-000494 | hsa-miR-22-000398 | hsa-miR-302d-000535 | hsa-miR-361-000554 | hsa-miR-433-001028 | hsa-miR-510-002241 | hsa-miR-526b-002382 | hsa-miR-567-001534 | hsa-miR-617-001591 | hsa-miR-664-002897 | hsa-miR-92a-1#-002137 |  |  |  |
| hsa-let-7g#-002118 | hsa-miR-1249-002868 | hsa-miR-1296-002908 | hsa-miR-151-3p-002254 | hsa-miR-196a#-002336 | hsa-miR-220b-002206 | hsa-miR-30a-3p-000416 | hsa-miR-361-3p-002116 | hsa-miR-448-001029 | hsa-miR-511-001111 | hsa-miR-532-001518 | hsa-miR-569-001536 | hsa-miR-618-001593 | hsa-miR-665-002681 | hsa-miR-92a-2#-002138 |  |  |  |
| hsa-let-7g-002282 | hsa-miR-1250-002887 | hsa-miR-1298-002861 | hsa-miR-151-5P-002642 | hsa-miR-196b-002215 | hsa-miR-220c-002211 | hsa-miR-30a-5p-000417 | hsa-miR-362-001273 | hsa-miR-449-001030 | hsa-miR-512-3p-001823 | hsa-miR-532-3p-002355 | hsa-miR-570-002347 | hsa-miR-620-002672 | hsa-miR-668-001992 | hsa-miR-92b#-002343 |  |  |  |
| hsa-let-7i#-002172 | hsa-miR-1251-002820 | hsa-miR-1300-002902 | hsa-miR-152-000475 | hsa-miR-197-000497 | hsa-miR-221#-002096 | hsa-miR-30b#-002129 | hsa-miR-362-3p-002117 | hsa-miR-449b-001608 | hsa-miR-512-5p-001145 | hsa-miR-539-001286 | hsa-miR-571-001613 | hsa-miR-621-001598 | hsa-miR-671-3p-002322 | hsa-miR-93#-002139 |  |  |  |
| hsa-miR-100#-002142 | hsa-miR-1252-002860 | hsa-miR-1301-002827 | hsa-miR-154#-000478 | hsa-miR-198-002273 | hsa-miR-221-000524 | hsa-miR-30b-000602 | hsa-miR-363#-001283 | hsa-miR-450a-002303 | hsa-miR-513-5p-002090 | hsa-miR-541#-002200 | hsa-miR-572-001614 | hsa-miR-622-001553 | hsa-miR-672-002327 | hsa-miR-933-002176 |  |  |  |
| hsa-miR-100-000437 | hsa-miR-1253-002894 | hsa-miR-1302-002901 | hsa-miR-154-000477 | hsa-miR-199a-000498 | hsa-miR-222#-002097 | hsa-miR-30c-000419 | hsa-miR-363-001271 | hsa-miR-450b-3p-002208 | hsa-miR-513B-002757 | hsa-miR-541-002201 | hsa-miR-573-001615 | hsa-miR-623-001555 | hsa-miR-674-002021 | hsa-miR-934-002177 |  |  |  |
| hsa-miR-1-002222 | hsa-miR-1254-002818 | hsa-miR-1303-002792 | hsa-miR-155#-002287 | hsa-miR-199a-3p-002304 | hsa-miR-222-002276 | hsa-miR-30c-1#-002108 | hsa-miR-365-001020 | hsa-miR-450b-5p-002207 | hsa-miR-513C-002756 | hsa-miR-542-3p-001284 | hsa-miR-574-3p-002349 | hsa-miR-624-001557 | hsa-miR-675-002005 | hsa-miR-935-002178 |  |  |  |
| hsa-miR-101#-002143 | hsa-miR-1255A-002805 | hsa-miR-1304-002874 | hsa-miR-155-002623 | hsa-miR-199b-000500 | hsa-miR-223#-002098 | hsa-miR-30c-2#-002110 | hsa-miR-367#-002121 | hsa-miR-452#-002330 | hsa-miR-515-3p-002369 | hsa-miR-542-5p-002240 | hsa-miR-575-001617 | hsa-miR-624-002430 | hsa-miR-708#-002342 | hsa-miR-936-002179 |  |  |  |
| hsa-miR-101-002253 | hsa-miR-1255B-002801 | hsa-miR-1305-002867 | hsa-miR-15a#-002419 | hsa-miR-19a#-002424 | hsa-miR-223-002295 | hsa-miR-30d#-002305 | hsa-miR-367-000555 | hsa-miR-452-002329 | hsa-miR-515-5p-001112 | hsa-miR-543-002376 | hsa-miR-576-3p-002351 | hsa-miR-625#-002432 | hsa-miR-708-002341 | hsa-miR-937-002180 |  |  |  |
| hsa-miR-103-000439 | hsa-miR-1256-002850 | hsa-miR-130a#-002131 | hsa-miR-15a-000389 | hsa-miR-19a-000395 | hsa-miR-224-002099 | hsa-miR-30d-000420 | hsa-miR-369-3p-000557 | hsa-miR-453-002318 | hsa-miR-516-3p-001149 | hsa-miR-544-002265 | hsa-miR-576-5p-002350 | hsa-miR-625-002431 | hsa-miR-7-2#-002314 | hsa-miR-938-002181 |  |  |  |
| hsa-miR-105#-002168 | hsa-miR-1257-002910 | hsa-miR-130a-000454 | hsa-miR-15b#-002173 | hsa-miR-19b-000396 | hsa-miR-23a#-002439 | hsa-miR-30e-3p-000422 | hsa-miR-369-5p-001021 | hsa-miR-454#-001996 | hsa-miR-516a-5p-002416 | hsa-miR-545#-002266 | hsa-miR-577-002675 | hsa-miR-626-001559 | hsa-miR-720-002895 | hsa-miR-939-002182 |  |  |  |
| hsa-miR-105-002167 | hsa-miR-1259-002796 | hsa-miR-130b#-002114 | hsa-miR-15b-000390 | hsa-miR-19b-1#-002425 | hsa-miR-23a-000399 | hsa-miR-31#-002113 | hsa-miR-370-002275 | hsa-miR-454-002323 | hsa-miR-516b-001150 | hsa-miR-545-002267 | hsa-miR-578-001619 | hsa-miR-627-001560 | hsa-miR-744#-002325 | hsa-miR-941-002183 |  |  |  |
| hsa-miR-106a#-002170 | hsa-miR-125a-3p-002199 | hsa-miR-130b-000456 | hsa-miR-16-000391 | hsa-miR-200a#-001011 | hsa-miR-23b#-002126 | hsa-miR-31-002279 | hsa-miR-371-3p-002124 | hsa-miR-455-001280 | hsa-miR-517#-001113 | hsa-miR-548a-001538 | hsa-miR-579-002398 | hsa-miR-628-3p-002434 | hsa-miR-744-002324 | hsa-miR-942-002187 |  |  |  |
| hsa-miR-106a-002169 | hsa-miR-125a-5p-002198 | hsa-miR-132#-002132 | hsa-miR-16-1#-002420 | hsa-miR-200a-000502 | hsa-miR-23b-000400 | hsa-miR-32#-002111 | hsa-miR-372-000560 | hsa-miR-455-3p-002244 | hsa-miR-517a-002402 | hsa-miR-548a-5p-002412 | hsa-miR-580-001621 | hsa-miR-628-5p-002433 | hsa-miR-758-001990 | hsa-miR-943-002188 |  |  |  |
| hsa-miR-106b#-002380 | hsa-miR-125b-000449 | hsa-miR-132-000457 | hsa-miR-16-2#-002171 | hsa-miR-200b#-002274 | hsa-miR-24-000402 | hsa-miR-320-002277 | hsa-miR-373-000561 | hsa-miR-483-3p-002339 | hsa-miR-517b-001152 | hsa-miR-548b-001541 | hsa-miR-581-001622 | hsa-miR-629-001562 | hsa-miR-765-002643 | hsa-miR-944-002189 |  |  |  |
| hsa-miR-106b-000442 | hsa-miR-125b-1#-002378 | hsa-miR-1324-002815 | hsa-miR-17#-002421 | hsa-miR-200b-002251 | hsa-miR-24-1#-002440 | hsa-miR-32-002109 | hsa-miR-374-000563 | hsa-miR-483-5p-002338 | hsa-miR-517c-001153 | hsa-miR-548b-5p-002408 | hsa-miR-582-3p-002399 | hsa-miR-629-002436 | hsa-miR-766-001986 | hsa-miR-95-000433 | | |  |
| hsa-miR-107-000443 | hsa-miR-125b-2#-002158 | hsa-miR-133a-002246 | hsa-miR-17-002308 | hsa-miR-200c#-002286 | hsa-miR-24-2#-002441 | hsa-miR-320B-002844 | hsa-miR-374a#-002125 | hsa-miR-484-001821 | hsa-miR-518a-3p-002397 | hsa-miR-548c-001590 | hsa-miR-582-5p-001983 | hsa-miR-630-001563 | hsa-miR-767-3p-001995 | hsa-miR-96#-002140 | |  |  |
| hsa-miR-10a#-002288 | hsa-miR-126#-000451 | hsa-miR-133b-002247 | hsa-miR-181a-000480 | hsa-miR-200c-002300 | hsa-miR-25#-002442 | hsa-miR-323-3p-002227 | hsa-miR-374b#-002391 | hsa-miR-485-3p-001277 | hsa-miR-518a-5p-002396 | hsa-miR-548c-5p-002429 | hsa-miR-583-001623 | hsa-miR-631-001564 | hsa-miR-767-5p-001993 | hsa-miR-98-000577 | | |  |
| hsa-miR-10a-000387 | hsa-miR-1260-002896 | hsa-miR-135a-000460 | hsa-miR-181a-2#-002317 | hsa-miR-202#-002362 | hsa-miR-25-000403 | hsa-miR-324-3p-002161 | hsa-miR-375-000564 | hsa-miR-485-5p-001036 | hsa-miR-518b-001156 | hsa-miR-548d-001605 | hsa-miR-584-001624 | hsa-miR-633-001574 | hsa-miR-769-3p-002003 | hsa-miR-99a#-002141 | |  |  |
| hsa-miR-10b#-002315 | hsa-miR-126-002228 | hsa-miR-135b#-002159 | hsa-miR-181c#-002333 | hsa-miR-202-002363 | hsa-miR-26a-000405 | hsa-miR-324-5p-000539 | hsa-miR-376a#-002127 | hsa-miR-486-001278 | hsa-miR-518c#-001158 | hsa-miR-548d-5p-002237 | hsa-miR-585-001625 | hsa-miR-634-001576 | hsa-miR-769-5p-001998 | hsa-miR-99a-000435 | |  |  |
| hsa-miR-10b-002218 | hsa-miR-1262-002852 | hsa-miR-135b-002261 | hsa-miR-181c-000482 | hsa-miR-203-000507 | hsa-miR-26a-1#-002443 | hsa-miR-325-000540 | hsa-miR-376a-000565 | hsa-miR-486-3p-002093 | hsa-miR-518c-002401 | hsa-miR-548E-002881 | hsa-miR-586-001539 | hsa-miR-635-001578 | hsa-miR-770-5p-002002 | hsa-miR-99b#-002196 | |  |  |
| hsa-miR-1178-002777 | hsa-miR-1263-002784 | hsa-miR-136#-002100 | hsa-miR-182#-000483 | hsa-miR-204-000508 | hsa-miR-26a-2#-002115 | hsa-miR-326-000542 | hsa-miR-376b-001102 | hsa-miR-487a-001279 | hsa-miR-518d-001159 | hsa-miR-548G-002879 | hsa-miR-587-001540 | hsa-miR-636-002088 | hsa-miR-802-002004 | hsa-miR-99b-000436 | |  |  |
| hsa-miR-1179-002776 | hsa-miR-1264-002799 | hsa-miR-136-000592 | hsa-miR-182-002334 | hsa-miR-205-000509 | hsa-miR-26b#-002444 | hsa-miR-328-000543 | hsa-miR-376c-002122 | hsa-miR-487b-001285 | hsa-miR-518d-5p-002389 | hsa-miR-548H-002816 | hsa-miR-588-001542 | hsa-miR-637-001581 | hsa-miR-871-002354 | mmu-let-7d#-001178 | |  |  |
| hsa-miR-1180-002847 | hsa-miR-1265-002790 | hsa-miR-138-002284 | hsa-miR-1825-002907 | hsa-miR-206-000510 | hsa-miR-26b-000407 | hsa-miR-329-001101 | hsa-miR-377#-002128 | hsa-miR-488-001106 | hsa-miR-518e#-002371 | hsa-miR-548I-002909 | hsa-miR-589-001543 | hsa-miR-638-001582 | hsa-miR-872-002264 | mmu-miR-124a-001182 | |  |  |
| hsa-miR-1182-002830 | hsa-miR-1267-002885 | hsa-miR-138-2#-002144 | hsa-miR-1826-002873 | hsa-miR-208-000511 | hsa-miR-27a#-002445 | hsa-miR-330-000544 | hsa-miR-377-000566 | hsa-miR-488-002357 | hsa-miR-518e-002395 | hsa-miR-548J-002783 | hsa-miR-589-002409 | hsa-miR-639-001583 | hsa-miR-873-002356 | mmu-miR-129-3p-001184 | |  |  |
| hsa-miR-1183-002841 | hsa-miR-1269-002789 | hsa-miR-139-3p-002313 | hsa-miR-183#-002270 | hsa-miR-208b-002290 | hsa-miR-27a-000408 | hsa-miR-330-5p-002230 | hsa-miR-378-000567 | hsa-miR-489-002358 | hsa-miR-518f#-002387 | hsa-miR-548K-002819 | hsa-miR-590-3P-002677 | hsa-miR-640-001584 | hsa-miR-874-002268 | mmu-miR-134-001186 | |  |  |
| hsa-miR-1184-002842 | hsa-miR-1270-002807 | hsa-miR-139-5p-002289 | hsa-miR-183-002269 | hsa-miR-20a#-002437 | hsa-miR-27b#-002174 | hsa-miR-331-000545 | hsa-miR-378-002243 | hsa-miR-490-001037 | hsa-miR-518f-002388 | hsa-miR-548L-002904 | hsa-miR-590-5p-001984 | hsa-miR-641-001585 | hsa-miR-875-3p-002204 | mmu-miR-137-001129 | |  |  |
| hsa-miR-1197-002810 | hsa-miR-127-000452 | hsa-miR-140-3p-002234 | hsa-miR-184-000485 | hsa-miR-20a-000580 | hsa-miR-27b-000409 | hsa-miR-331-5p-002233 | hsa-miR-380-3p-000569 | hsa-miR-491-3p-002360 | hsa-miR-519a-002415 | hsa-miR-548M-002775 | hsa-miR-591-001545 | hsa-miR-642-001592 | hsa-miR-875-5p-002203 | mmu-miR-140-001187 | |  |  |
| hsa-miR-1200-002829 | hsa-miR-1271-002779 | hsa-miR-141#-002145 | hsa-miR-185#-002104 | hsa-miR-20b#-002311 | hsa-miR-28-000411 | hsa-miR-335#-002185 | hsa-miR-380-5p-000570 | hsa-miR-492-001039 | hsa-miR-519b-3p-002384 | hsa-miR-548N-002888 | hsa-miR-592-001546 | hsa-miR-643-001594 | hsa-miR-876-3p-002225 | mmu-miR-153-001191 | |  |  |
| hsa-miR-1201-002781 | hsa-miR-1272-002845 | hsa-miR-141-000463 | hsa-miR-185-002271 | hsa-miR-20b-001014 | hsa-miR-28-3p-002446 | hsa-miR-335-000546 | hsa-miR-381-000571 | hsa-miR-493-002364 | hsa-miR-519c-001163 | hsa-miR-548P-002798 | hsa-miR-593-001547 | hsa-miR-644-001596 | hsa-miR-876-5p-002205 | mmu-miR-187-001193 | |  |  |
| hsa-miR-1203-002877 | hsa-miR-1274A-002883 | hsa-miR-142-3p-000464 | hsa-miR-186#-002105 | hsa-miR-21#-002438 | hsa-miR-296-000527 | hsa-miR-337-3p-002157 | hsa-miR-382-000572 | hsa-miR-494-002365 | hsa-miR-519d-002403 | hsa-miR-549-001511 | hsa-miR-593-002411 | hsa-miR-645-001597 | hsa-miR-885-3p-002372 | mmu-miR-374-5p-001319 | |  |  |
| hsa-miR-1204-002872 | hsa-miR-1274B-002884 | hsa-miR-142-5p-002248 | hsa-miR-186-002285 | hsa-miR-210-000512 | hsa-miR-296-3p-002101 | hsa-miR-337-5p-002156 | hsa-miR-383-000573 | hsa-miR-497#-002368 | hsa-miR-519e#-001166 | hsa-miR-550-001544 | hsa-miR-595-001987 | hsa-miR-646-001599 | hsa-miR-885-5p-002296 | mmu-miR-379-001138 | |  |  |
| hsa-miR-1205-002778 | hsa-miR-1275-002840 | hsa-miR-143#-002146 | hsa-miR-188-3p-002106 | hsa-miR-21-000397 | hsa-miR-298-002190 | hsa-miR-338-3p-002252 | hsa-miR-384-000574 | hsa-miR-497-001043 | hsa-miR-519e-002370 | hsa-miR-550-002410 | hsa-miR-596-001550 | hsa-miR-647-001600 | hsa-miR-886-3p-002194 | mmu-miR-451-001141 | |  |  |
| hsa-miR-1206-002878 | hsa-miR-127-5p-002229 | hsa-miR-143-002249 | hsa-miR-18a#-002423 | hsa-miR-211-000514 | hsa-miR-299-3p-001015 | hsa-miR-338-5P-002658 | hsa-miR-409-3p-002332 | hsa-miR-499-3p-002427 | hsa-miR-520a#-001168 | hsa-miR-551a-001519 | hsa-miR-597-001551 | hsa-miR-648-001601 | hsa-miR-886-5p-002193 | mmu-miR-491-001630 | |  |  |
| hsa-miR-1208-002880 | hsa-miR-1276-002843 | hsa-miR-144#-002148 | hsa-miR-18a-002422 | hsa-miR-212-000515 | hsa-miR-299-5p-000600 | hsa-miR-339-3p-002184 | hsa-miR-409-5p-002331 | hsa-miR-500-001046 | hsa-miR-520a-001167 | hsa-miR-551b#-002346 | hsa-miR-598-001988 | hsa-miR-649-001602 | hsa-miR-887-002374 | mmu-miR-495-001663 | |  |  |
| hsa-miR-122#-002130 | hsa-miR-1278-002851 | hsa-miR-144-002676 | hsa-miR-18b#-002310 | hsa-miR-213-000516 | hsa-miR-29a#-002447 | hsa-miR-339-5p-002257 | hsa-miR-410-001274 | hsa-miR-500-002428 | hsa-miR-520b-001116 | hsa-miR-551b-001535 | hsa-miR-599-001554 | hsa-miR-650-001603 | hsa-miR-888#-002213 | mmu-miR-496-001953 | |  |  |
| hsa-miR-122-002245 | hsa-miR-1282-002803 | hsa-miR-145#-002149 | hsa-miR-18b-002217 | hsa-miR-214#-002293 | hsa-miR-29a-002112 | hsa-miR-33a#-002136 | hsa-miR-411#-002238 | hsa-miR-501-001047 | hsa-miR-520c-3p-002400 | hsa-miR-552-001520 | hsa-miR-600-001556 | hsa-miR-651-001604 | hsa-miR-888-002212 | mmu-miR-499-001352 | |  |  |
