# Supplementary material for: UVA influenced the SIRT1‐miR‐27a‐5p‐SMAD2‐MMP1/COL1/BCL2 axis in human skin primary fibroblasts
Source: J Cell Mol Med. 2020 Aug 13;24(17):10027–41. doi: 10.1111/jcmm.15610 (PMC7520305; doi:10.1111/jcmm.15610)
Supplement: Supplementary file 3 — Table S2 [file JCMM-24-10027-s003.docx]

Supplement Table2 The target genes of miR-27a-5p.

| ABAT | ABCA1 | abcb10 | ABCC4 | ABCF2 | ABCG1 | | ABHD13 | | ABLIM1 | ABTB2 | | ACVR2A |
| --- | --- | --- | --- | --- | --- | --- | --- | --- | --- | --- | --- | --- |
| ACVR2B | ADAM19 | ADAM9 | ADAMTS17 | ADAMTS5 | ADAMTS6 | | ADCY6 | | ADCY9 | ADD1 | | AEN |
| AFF2 | Ago1 | AGPAT4 | AK2 | AK9 | AKR7A2 | | AKT3 | | AMD1 | AMMECR1 | | AMMECR1L |
| AMOT | ANK3 | ANKFY1 | ANKRD13C | ANKRD44 | ANKRD52 | | ANTXR2 | | AP1G1 | APBA2 | | APBB2 |
| APBB3 | APEX1 | APEX2 | apln | APLNR | APOO | | APPBP2 | | AQP4 | ARC | | ARF3 |
| ARF6 | ARFGEF1 | ARFGEF2 | ARHGAP28 | ARHGAP5 | ARID1A | | ARID4A | | ARL3 | ARL5B | | ARMC8 |
| ARPP19 | ARRDC3 | ARX | ASB14 | ASB15 | ASB6 | | ASF1A | | ASH2L | atg2b | | ATG4B |
| ATG9A | ATM | ATP11C | ATP2B1 | ATP2B2 | ATP2B4 | | ATP9A | | ATXN3 | ATXN7L1 | | AXIN2 |
| AZIN1 | BACE1 | BACH2 | BAIAP2 | BCAS3 | BCAT1 | | BCL2 | | BCO2 | BDNF | | BEND4 |
| BICD2 | BMF | BMI1 | BMPR1A | BMS1 | BNC2 | | BPTF | | BRD1 | BRD4 | | BRINP2 |
| BRPF3 | BRWD1 | BSDC1 | BTBD7 | BTF3 | BTG1 | | BTN2A1 | | BTN2A2 | BUB3 | | C11orf30 |
| C15orf27 | C1QL3 | C22orf23 | C2orf88 | c6orf106 | C6orf120 | | CA8 | | CAB39 | CACNA1C | | CACNA1I |
| CACNA2D2 | CALML4 | CALN1 | CAMTA1 | CASP8 | CCDC6 | | CCDC71L | | CCDC81 | CCND3 | | CCNJ |
| CCS | CD1C | CD1D | CD59 | CDC14B | CDC42 | | CDC42BPA | | CDC5L | CDCP1 | | CDK19 |
| CDK5 | CDK6 | CEACAM1 | CELF2 | CELSR3 | CENPP | | CEP170 | | CEPT1 | CHD5 | | CHD7 |
| CHERP | CHM | CHSY1 | CHTF8 | CHURC1 | CKAP5 | | CLIP3 | | CLLU1 | CLOCK | | CNBP |
| CNOT1 | CNOT7 | CNR1 | CNTN3 | CNTN4 | COBLL1 | | COL12A1 | | COL4A3BP | COL9A1 | | COL9A3 |
| COMMD9 | COPS7B | CPNE8 | CREB3L2 | CREB5 | CREBBP | | CREM | | CRKL | CRTC1 | | CSMD2 |
| CSNK1G1 | CSNK1G3 | CTBP2 | CTCF | CTDSP2 | CTNNA3 | | CTNNB1 | | CTR9 | CYFIP2 | | CYP7B1 |
| DAZAP2 | DAZL | DCAF4 | DCAF5 | DCAF7 | DCP1A | | DCP2 | | DCUN1D3 | DCX | | DDAH1 |
| DDX3Y | DDX6 | DENND4C | DEPDC1 | DERL2 | DGCR2 | | DGKB | | DIEXF | DIP2C | | DLG2 |
| DLGAP2 | DMBX1 | DMD | DMGDH | DMXL1 | DNAJA2 | | DNAJB12 | | DNAJB4 | DNAJB5 | | DNAJC16 |
| DNALI1 | DNM3 | DNPEP | DPP10 | DPY19L2 | DPYD | | DR1 | | DSC3 | DUSP3 | | DYNC1LI2 |
| DYRK2 | EBAG9 | EDEM3 | EDF1 | EDNRB | EGR3 | | EI24 | | EID1 | EIF1AX | | EIF5 |
| EIF5B | ELAVL1 | ELFN2 | ELL2 | ELMO1 | ELMOD1 | | ELOVL5 | | ELOVL6 | EMC8 | | ENPEP |
| EP400 | EPB41 | EPB41L5 | EPHB2 | ERBB3 | ERBB4 | | ERF | | ERG | ERGIC2 | | ERLIN1 |
| ERP44 | ESYT2 | ETV1 | ETV6 | EVL | EXOC4 | | EXOC8 | | EYA4 | F13A1 | | FADS1 |
| FAM107B | FAM118B | FAM120A | FAM120C | FAM135A | FAM222B | | FAM45A | | FAM46A | FAM53C | | FBLN1 |
| FBXL18 | FBXL4 | FBXL7 | FBXO11 | FBXO28 | FBXO33 | | FBXO45 | | FBXO8 | FBXW11 | | FBXW2 |
| FCRL1 | FGD6 | FGFR2 | FKBP1A | FLI1 | FLRT3 | | FN1 | | FOXG1 | FOXN1 | | FOXN3 |
| FOXO1 | FOXP1 | FOXP4 | FRA10AC1 | FSD2 | FSTL4 | | FXR1 | | FZD5 | G3BP1 | | GAB2 |
| GABRA4 | GALNT3 | GALNT6 | GAPVD1 | GATAD2B | GBF1 | | GFPT2 | | GFRA1 | GJA3 | | GK |
| GLI3 | GLRA3 | GLTSCR1L | GMEB2 | GNA12 | GNA13 | | GNG4 | | GNL3L | GPATCH2 | | GPR158 |
| GPR173 | GPR180 | GPR6 | GPR68 | GPRC5B | GRB2 | | GRIK2 | | GRIK3 | GRIN2B | | GRM4 |
| GRSF1 | GSPT1 | GTPBP1 | H3F3B | HAPLN1 | HAUS3 | | HBP1 | | HDAC4 | HDHD2 | | HECW1 |
| HECW2 | HES1 | HFM1 | HGS | HIVEP3 | HK1 | | HLF | | HMGN2 | HNF1B | | HOXB13 |
| HOXB7 | HP1BP3 | HRH1 | HS2ST1 | HS6ST3 | HSDL2 | | HSPH1 | | IBA57 | IBTK | | ICA1L |
| IFFO2 | IGF2BP3 | IGFBP5 | IHH | IL1R1 | IL2 | | INO80 | | INPP5B | INTS6 | | INTS7 |
| IP6K1 | IPO9 | IPPK | IREB2 | ITCH | ITGA11 | | ITPKB | | ITPR2 | ITSN1 | | JMJD1C |
| JPH2 | KALRN | KANK1 | KATNBL1 | KCMF1 | KCNAB2 | | KCNC1 | | KCND3 | KCNJ16 | | KCNK1 |
| KCTD12 | KDM3B | KDM5A | KDM5C | KHDRBS1 | KIAA0895 | | KIAA1009 | | KIAA1324 | KIAA1324L | | KIAA1958 |
| KIF1B | KIF21B | KIF3B | KLHL28 | KLHL29 | KLHL3 | | KLHL42 | | KMT2A | KPNA4 | | KPNA6 |
| KREMEN1 | L2HGDH | LAMA4 | LARP1 | LARP4 | LCOR | | LDB1 | | LDLRAP1 | LEF1 | | LHFPL4 |
| LHX8 | LHX9 | LIFR | LIMD2 | LIN28B | LIX1 | | LMBR1 | | LMNB1 | LPGAT1 | | LPP |
| LRAT | LRP8 | LRRC7 | LSM12 | LTBP1 | LUZP1 | | LY75 | | LYRM2 | LYRM4 | | LZTFL1 |
| MACF1 | MAN1A2 | mansc1 | MAP1B | MAP2K2 | MAP3K7 | | MAPK1 | | MAPK6 | MAPKBP1 | | MAPRE2 |
| MAPT |  | MASP1 | MAX | MBD2 | MBNL3 | | MBTD1 | | MCM10 | MCU | | MDGA1 |
| MDGA2 | MDN1 | ME3 | MECP2 | MED1 | MED12L | | MED22 | | MED29 | MEF2D | | MEIS1 |
| MELK | MEOX2 | METTL10 | METTL13 | METTL8 | MFAP1 | | MFN1 | | MFSD8 | MINA | | MIXL1 |
| MKL2 | MLLT10 | MLLT3 | MLLT6 | MMAB | MOB3A | | MORC3 | | MOSPD1 | MPPED2 | | MRGBP |
| MST4 | MTF1 | MTHFR | MTMR3 | MTPN | MTRNR2L9 | | MTSS1 | | MTX3 | MXI1 | | MYO1E |
| MYO6 | MYPN | NAALADL2 | NADK | NALCN | NAMPT | | NANOS1 | | NAP1L5 | NAPA | | NAV3 |
| NCAM1 | NCKAP1 | NCOA1 | NCOA2 | NEGR1 | NEO1 | | NETO1 | | NETO2 | NEUROD6 | | NF2 |
| NFAM1 | NFASC | NFAT5 | NFE2L1 | NFIB | NFIC | | NHLRC3 | | NHS | NIN | | NIPAL2 |
| NKIRAS1 | NMNAT2 | NNT | NOTCH1 | NOTCH2 | NOVA1 | | NOX1 | | NPM1 | NPTX1 | | NR2F2 |
| NRIP3 | NRP2 | NT5C2 | NT5E | NUFIP2 | NUP133 | | OSR2 | | OTUD3 | OTUD7A | | OTUD7B |
| PADI2 | PAF1 | PAG1 | PAIP2 | PAK1 | PAK3 | | PALM2 | | PANK1 | PANK4 | | PAPPA |
| PARK2 | PARM1 | PARVA | PATZ1 | PAWR | PAX6 | | PAX8 | | PBOV1 | PCDH1 | | PCDH17 |
| PCDH20 | PCDHA1 | PCDHA11 | PCDHA12 | PCDHA2 | PCDHA3 | | PCDHA4 | | PCDHA5 | PCDHA6 | | PCDHA7 |
| PCDHA8 | PCDHA9 | PCGF3 | PCM1 | PCMTD1 | PCNP | | PCNXL2 | | PCYT1A | PDE3A | | PDE4D |
| PDE4DIP | PDE5A | PDE8A | PDGFA | PELI1 | PER2 | | PEX6 | | PHF21A | PHF8 | | PHLDB1 |
| PIGK | PIK3CG | PJA2 | PKN2 | PKP4 | PLAGL2 | | PLCE1 | | PLCL1 | PLEKHA6 | | PLEKHM1 |
| plxdc2 | plxna1 | PMEPA1 | PNRC2 | POLR1D | pom121 | | PON2 | | POSTN | PPIL4 | | PPM1A |
| PPME1 | PPP1R12B | PPP2R2A | PPP4R1 | PPP4R4 | PPP6C | | PRDM1 | | PRDM16 | PRIMA1 | | PRKAB2 |
| PRKCDBP | PRKG1 | PRKG2 | PRLR | PRPF19 | PRPS1 | | PRR16 | | PRRX2 | PRX | | PSAT1 |
| PSMA1 | PSME1 | PTBP2 | PTCH2 | PTCHD1 | PTP4A2 | | PTPN11 | | PTPRB | PTPRF | | PTPRJ |
| PTPRT | PURA | PXMP4 | QKI | QRICH1 | R3HDM1 | | RAB3IP | | RAB43 | RAB6A | | RAB6C |
| RABGAP1 | RAD23B | RAD50 | RALGAPB | RALGPS1 | RANBP6 | | RAP2A | | RASA3 | RASEF | | RASGRP1 |
| RASL12 | RASSF2 | RASSF5 | RBBP6 | RBM15 | RBM24 | | RBM33 | | RBM39 | RBM8A | | RBMS3 |
| RCBTB1 | RELN | REPS2 | RFK | RFX3 | RGMA | | RGS4 | | RGS7BP | RIMS4 | | RMND5B |
| rnaseh2c | RND2 | RNF14 | RNF144A | RNF150 | RNF152 | | RNF214 | | RNF38 | RNF44 | | RPS24 |
| RPS6KA3 | RPS6KA5 | RRP1B | RS1 | RSBN1 | RSPO2 | | RTKN2 | | RTN4 | RTTN | | RUNX1 |
| RYBP | RYK | SALL3 | SAMD12 | SAP18 | SCAMP1 | | SCN8A | | SCP2 | SCYL3 | | SDCBP |
| SDK2 | SEC22A | SEMA5A | SEMA6A | SEMA6D | SERBP1 | SERINC1 | | SERPINB3 | | |  |  |
| SERPINB4 | SESN1 | SESTD1 | SF3A1 | SF3A3 | SGTA | | SGTB | | SH2B3 | SH2D3C | | SH3KBP1 |
| SHANK2 | SHB | SHISA9 | SHKBP1 | SKP1 | SLC12A6 | | SLC1A2 | | SLC23A2 | SLC25A53 | | SLC2A1 |
| SLC2A8 | SLC31A1 | SLC35B4 | SLC35F1 | SLC40A1 | SLC44A1 | | SLC4A4 | | SLC8A1 | SLCO5A1 | | SLITRK1 |
| SLITRK2 | SMAD2 | SMAD4 | SMAP1 | SMARCA1 | SMG5 | | SMG7 | | SMURF2 | SNN | | SNX12 |
| SNX27 | SOBP | SOD2 | sorbs1 | SORBS2 | SORCS1 | | SOSTDC1 | | SOX1 | SOX5 | | SP1 |
| SP2 | SP6 | SPICE1 | SPIN1 | SPPL3 | SPTLC2 | | SPTLC3 | | SREK1 | SRGAP2 | | SRGAP3 |
| SRR | SRSF1 | SRSF10 | SS18L1 | SSFA2 | ST5 | | ST8SIA1 | | ST8SIA4 | STAT3 | | STIM2 |
| STK39 | STK4 | STOML1 | STON1 | STRBP | STRN4 | | STXBP4 | | SUCO | SUMO1 | | SYNJ1 |
| SYPL1 | SYT7 | TACC1 | TAL1 | TANC1 | TAOK1 | | TASP1 | | TBC1D25 | TBK1 | | TBL1XR1 |
| TBPL1 | TCEB3 | TCERG1L | TEAD1 | TENM1 | TET1 | | TFCP2 | | TFDP1 | TGFBR2 | | TGIF1 |
| THNSL1 | THRB | THSD4 | TIPARP | TJP2 | TLE4 | | TLK2 | | TLL2 | TM2D2 | | TMCC1 |
| TMCC3 | TMEM106B | TMEM158 | TMEM181 | TMEM242 | TMEM33 | | TMEM39B | | TMEM50B | TMTC2 | | TNPO1 |
| TNPO3 | TNR | TP53INP2 | TPM3 | TRAM2 | TRAPPC8 | | TRIM24 | | TRIM25 | TRIM67 | | TRMT5 |
| TSHZ3 | TSPAN14 | TSPAN6 | TSSK1B | TTBK2 | TTC14 | | ttc30a | | TTLL7 | TUSC3 | | UAP1 |
| UBE2D3 | UBE2N | UBE2W | UBE2Z | UBE3C | UBXN6 | | UNC13C | | UPF3B | USP2 | | USP21 |
| USP31 | USP47 | USP9X | UTRN | UVRAG | VAMP1 | | VANGL1 | | VAV3 | VCL | | VEGFC |
| VEZF1 | VGLL2 | VPS13B | VPS26A | VSNL1 | VTI1A | | WAC | | WBP1L | WDFY3 | | WDR26 |
| WDR48 | WDR89 | WHSC1 | WNK3 | WNT4 | WT1 | | XPO4 | | XRCC5 | XYLT1 | | YWHAB |
| YWHAQ | ZADH2 | ZBTB10 | ZBTB24 | ZBTB37 | ZBTB39 | | ZBTB4 | | ZBTB41 | ZBTB44 | | ZC3H15 |
| ZCCHC24 | ZCCHC8 | ZDHHC17 | ZDHHC18 | ZDHHC3 | ZDHHC9 | | ZEB1 | | ZFP36L2 | ZFP91 | | ZFX |
| ZFYVE1 | ZHX1 | ZMAT1 | ZMIZ1 | ZMPSTE24 | ZMYM1 | | ZMYM2 | | ZMYM4 | ZNF106 | | ZNF281 |
| ZNF3 | ZNF326 | ZNF366 | ZNF384 | ZNF410 | ZNF436 | | ZNF512B | | ZNF544 | ZNF608 | | ZNF621 |
| ZNF623 | ZNF652 | ZNF704 | ZNF800 | ZRANB3 | ZZZ3 | |  | |  |  | |  |
